# Kissing Bug Intrusions into Homes in the Southwest United States

**DOI:** 10.3390/insects12070654

**Published:** 2021-07-17

**Authors:** Stephen A. Klotz, Shannon L. Smith, Justin O. Schmidt

**Affiliations:** 1 Division of Infectious Diseases, Department of Medicine and Family and Community Medicine, University of Arizona, Tucson, AZ 85724, USA; shannonlsmith814@gmail.com; 2 Southwestern Biological Institute and Department of Entomology, University of Arizona, Tucson, AZ 85721, USA; ponerine@dakotacom.net

**Keywords:** kissing bug, insect bites, Chagas disease, *Trypanosoma cruzi*, allergic reactions to insect bites, *Triatoma rubida*, *Triatoma protracta*

## Abstract

**Simple Summary:**

Kissing bugs are a nuisance insect in the Desert Southwest of the United States. As people build their homes in kissing bug habitats, bugs frequently enter the homes to obtain a blood meal from the human and pet inhabitants. Proteins in bug saliva can cause severe allergic reactions, although the bite itself is painless. Many kissing bugs harbor the parasite, *Trypanosoma cruzi*, in their gastrointestinal tract and pass it in their feces. This parasite causes Chagas disease in humans and many mammals, particularly in Central and South America. Fortunately, few human infections occur in the US. In this article, we investigate homes that kissing bugs have entered in the Southwest and attempt to determine the risk this poses for homeowners for infection and measures that can be taken to reduce intrusion.

**Abstract:**

Kissing bugs readily enter homes in the Sonoran Desert and bite the residents. Their saliva is highly antigenic, causing local and systemic skin reactions and life-threatening anaphylaxis. We attempted to determine what characteristics of homesites may have contributed to home intrusion by kissing bugs. Extensive and detailed information about the homes and the home environment was collected from 78 homeowners in Tucson who suffered kissing bug intrusions. Homeowners collected 298 *Triatoma rubida* in and around their homes. Of the homes entered by kissing bugs, 29 of 46 (63%) contained bugs harboring *Trypanosoma cruzi*. Although in the aggregate, homeowners were bitten > 2200 times, no individual tested positive for Chagas disease (N = 116). Although yearly intrusion likely occurs in some homes, *T. rubida* does not domiciliate within homesites in the Desert Southwest. We conclude there is little risk to homeowners for Chagas disease given the current behavior of resident kissing bugs and absent ingesting kissing bug fecal matter.

## 1. Introduction

*Triatoma infestans*, historically the most important kissing bug in South America, is almost entirely domesticated, and until recently only a few wild type populations were thought to survive in Bolivia [1]. Historically, domiciles constructed with wattle and daub walls and palm-thatched roofing provided abundant refugia for the insects. Thousands of bugs lived within these homes, and eventually the residents became infected with *Trypanosoma cruzi* [2]. These types of houses are not so common anymore. Interestingly, oral ingestion of the parasite as a mode of infection is being recognized throughout South America [3].

The authors of this communication reside in Tucson, Arizona, situated in the Sonoran Desert, a geographic region rich in numbers and species of kissing bugs. The most populous kissing bug, *Triatoma rubida*, makes its abode within nests of packrats (*Neotoma albigula)* that abound due to the presence of prickly pear (*Opuntia* spp.) and cholla cacti (*Cylindropuntia* spp.) plants that provide food, water and shelter for the rodents. The packrat is the most numerous animal reservoir of *T. cruzi* in the Sonoran Desert as first noted in Arizona by S.F. Wood [4]. In Tucson, roughly 50% of wild packrats tested are positive for *T. cruzi* by blood smear (personal communication, C. Sterling). Other woodrat species are reservoirs of *T. cruzi* in California where *T. protracta* is the most common triatome. Annually in the months of May and June in the Sonoran Desert, *T. rubida* undergoes flight dispersals in the early evening hours, and attracted by outside house lights, roaming kissing bugs may enter domiciles in the foothills surrounding Tucson [5].

In the United States where Chagas disease is rare, it is often stated that houses are less susceptible to kissing bug intrusion because homes are generally newer, feature air conditioning, window screening, and possess solid foundations and walls [6]. As we have had a longstanding interest in home intrusion by kissing bugs in the Southwest, we present evidence and discuss the relationship, if any, between home construction and the home environment and kissing bug intrusion. Homeowners who experienced kissing bug intrusions provided information for a detailed peri-domiciliary questionnaire (Supplementary Data 1) that investigated properties of the home, outbuildings, household members, pets and livestock, environment and landscaping around the home. A separate survey tested homeowner knowledge of kissing bug identification, information surrounding bites and the reaction to bites (Supplementary Data 2). These factors and others were used to generalize about the risk for intrusion. Performing serological testing of homeowners and household members allowed us to make some estimate of the risk for acquisition of Chagas disease and its relation to kissing bug home intrusion [7].

## 2. Materials and Methods

In this report, we provide information from what we term public interactions (explained below) as well as data from a structured research study, some of which has been presented elsewhere [7].

Public interactions other than study participants. Residents of the Sonoran Desert regularly call or email our research team and local health officials throughout the year, especially in late May and June, when kissing bugs disperse from their native habitat and enter households. In addition, we are known as kissing bug “experts” from television, public speaking, newspapers, University of Arizona blogs and publications where our work telephone number and email addresses can be found. Public interactions were entered as results where pertinent and appropriate.

Tucson home study. The home study occurred from April 2017 through October 2018 mainly in Tucson, AZ [7]. Homeowners (N = 78) whose residence was invaded by kissing bugs provided informed consent to the study approved by the University of Arizona Institutional Review Board. The IRB forbade contacting homeowners directly, so we were limited to homeowners who contacted us. Consequently, a proper random survey of a community could not be performed. However, in Tucson we devoted months to putting on public forums to enroll homeowners on site. Each participating homeowner was interviewed and a survey filled out (Supplementary Data 1).

Kissing bugs and *Trypanosoma cruzi* detection. Before entering the study homeowners provided live or dead kissing bugs for morphological identification and PCR testing of intestinal contents for *T. cruzi*. They received written instructions on how to collect and safely handle kissing bugs along with cups with screw caps and nitrile gloves and filled out a household survey (Supplementary Data 1) as well as their knowledge of kissing bugs and bite history (Supplementary Data 2). Captured kissing bugs were brought in sealed plastic bags, removed from the bag and stored in tubes with 100% ETOH at −20 °C. To ensure that no ETOH was present during dissection, samples were washed with sterile water and dried thoroughly prior to dissection. The innards of the abdominal fragment of each bug were scraped from the walls of the exoskeleton and added to a sterile tube for DNA extraction. DNA extraction was performed using Qiagen DNeasy Blood and Tissue kit following the manufacturer’s recommendations (Qiagen DNeasy, Hilden, Germany) [7]. Carriage of *T. cruzi* by the kissing bugs was determined by PCR, amplifying a 188-bp segment of the 195-bp repetitive nuclear sequence of *T. cruzi* as explained in detail previously [7].

Chagas tests. Houseowners with verified kissing bug intrusion and bites were tested for Chagas disease using Chagas Detect Plus Rapid Test (InBios, Seattle, WA, USA) [7]. Trained staff performed the tests and questionable tests were repeated. Indeterminate results were resolved by sending serum to the Centers for Disease Control and Prevention (CDC), Atlanta, GA for confirmation. The confirmation tests included EIA recombinant 3.0 (an enzyme immunoassay from Weiner Lab, Rosario, Argentina) and Trypomastigote Excreted-Secreted Antigen or TESA (bioMerieux, Buenos Aires, Argentina).

## 3. Results

Public interactions other than with home study participants. May 2020 was a remarkable month for dispersing kissing bugs (For example, one author (JOS) captured 335 *T. rubida* and *T. protracta* at his homesite in May and June 2020). We received daily calls and emails from the public to retrieve kissing bugs from individuals who had captured them in their homes. People’s interest in these insects has undergone a change. For example, a decade ago, the public phoned or visited the clinic because of allergies to kissing bug bites, including anaphylaxis whereas, today’s callers usually contact us to request blood tests for Chagas disease. Photographs of bite reactions are often attached to emails (see below). Our responses to phone calls, emails and clinic visits stress the unlikelihood of infection with *T. cruzi* and do not recommend obtaining commercially available serological tests. Nevertheless, about 10% of callers continue to call until they do obtain such a test. We have never had a positive serology for Chagas disease in either our clinical or research work, representing hundreds of requested serological tests. However, false-positive tests requiring submission of serum to the Centers of Disease Control for confirmation do occur, and several occurred in our recent home study [7].

Some representative emails with attached photos are shown below. We receive emails like these every several months, along with questions of what to do and whether or not a serology for Chagas disease should be obtained. We discourage people obtaining serological tests because the commercially available tests are insensitive and have an unknown rate of false-positive results.

**Figure 1:**
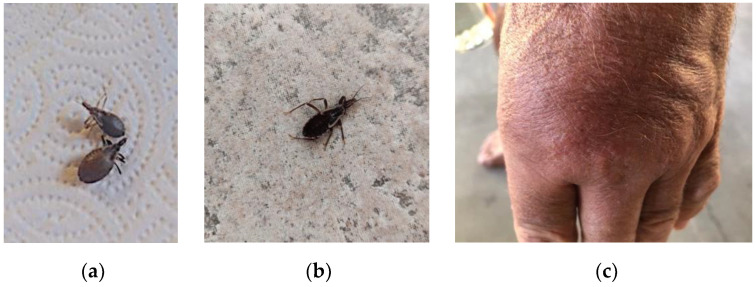
(**a**) Two replete nymphs, likely 2nd and 3rd stage nymphs of *T. rubida*. (Note, these were found in December.) (**b**) A 4th stage nymph of *T. rubida* on the bedroom wall in June. (**c**) Swollen and erythematous dorsum of the left hand after feeding by *T. rubida* in June.

**Example** **1.** *“My husband and I were in our bedroom collecting old clothes for the Salvation Army. What appeared to be a well-fed kissing bug fell from a shirt. I collected it in a water bottle. Then about 15 min later we discovered another one… Not as well fed.” (email, 16 December 2016; Figure 1a). This occurred in Tucson, AZ, age of home unknown.*

**Example** **2.** *“pics of the two that I found inside my house, one on the kitchen wall and the other on the wall going up the stairwell…” (email, 4 June 2020; Figure 1b). The house is a new construction in Oracle, AZ.*

**Example** **3.** *77-year-old man bitten at night complains of considerable swelling on the dorsum of left hand where there were several puncta (email, 30 June 2020; Figure 1c). Serologies were demanded, performed and were negative. This occurred in an older home in Rio Rico, AZ.*

Tucson home study. Homes included in the study in Tucson were for the most part of recent construction, the median age being 7 years as opposed to the town of Bisbee, AZ where the median age of homes was 91 years [7]. Tucson homes were constructed on solid concrete slab foundations and 79.2% of homes used air conditioning; a third used pesticides in the home and roughly a fifth, used a pest control service [7]. Bugs were discovered inside the homes and brought for analysis in 44 of 74 total homes in the Tucson study. Homeowners turned in 298 kissing bugs captured in or around their homes, all were *T. rubida* and the majority were adults, but 13 homes had females with eggs; and nine homes had nymphs (Table 1). Although 63% (29 of 46) homes were invaded by kissing bugs harboring *T. cruzi* (Table 1) and the residents of the homes were bitten more than 2200 times in the aggregate, no resident had evidence of Chagas disease by serological tests [7].

**Figure 2:**
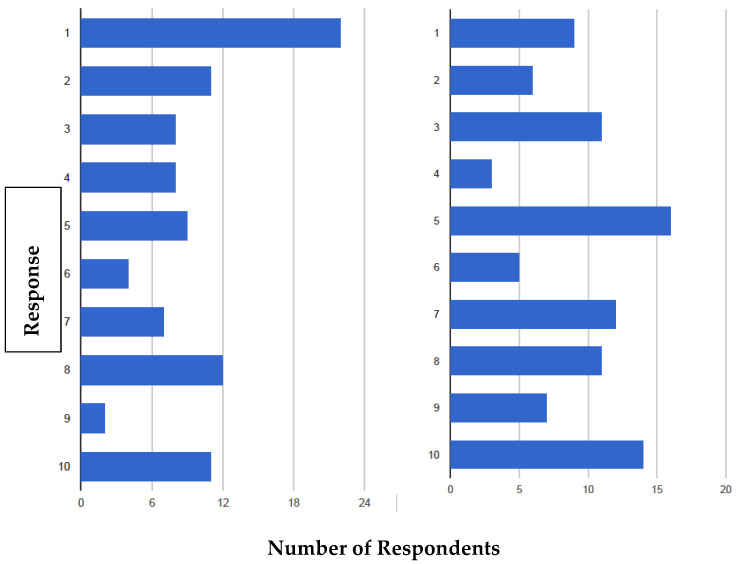
Fear and frustration with kissing bugs. (**Left**). Homeowners were asked “How afraid of kissing bugs are you?” (scale of 1–10; 1, being not afraid and 10, being most afraid). (**Right**). Homeowners were asked: “Knowing kissing bugs bite at night, how safe do you feel when sleeping?” (scale of 1–10; 1, feeling safe and 10, feeling not safe at all). N = 91 for both surveys.

Entry of kissing bugs into a home often induces pandemonium within the household. Some homeowners fear being bitten by kissing bugs at night and adopt unusual sleeping habits: using mosquito netting or constructing cloth sleeping sacks designed to reduce exposure of the skin. This behavior is driven by fear that they will contract Chagas disease from the bites, which is clearly not the case [8,9]. However, this fear is impossible to dispel in many bite victims. In any case, we attempted to determine the levels of concern about being bitten by kissing bugs (Figure 2).

It is apparent from Figure 2 (left) that 27.4% (25) of the respondents were afraid of kissing bugs (response, 8–10) and Figure 2 (right) shows 39.5% (36) did not feel safe when sleeping.

We spend considerable time discussing callers’ domiciles and efforts to stop kissing bug entry [10]. In addition, we send most callers and Teleconsult patients a copy of an easily understood pamphlet about kissing bugs that explains some simple measures homeowners may take to reduce the likelihood of kissing bug intrusion [11].

Pets and Livestock. Of the many characteristics of the homes we studied, the presence of pets and/or other animals in the peridomestic area could be an inducement for kissing bugs to favor a particular structure. Fifty-three homeowners (69% of homes) claimed to have pets, mainly dogs or cats (Table 1). Some homeowners had more than one cat or dog and some had both. In houses that reported having pets, seven (18%) reported having >2 dogs in the home and nine (31%) reported having >2 cats in the home. Of the three participants who raised cattle, all reported having >15 on the property and, similarly, three of the nine participants who had horses reported having >15 on the property.

## 4. Discussion

After more than 20 years of observation and studying home intrusion by kissing bugs in the Sonoran Desert, we are confident we can explain some of the “whys and wherefores” of kissing bug entry into homes.

If you build a home in kissing bug habitat, expect your home to be entered by these bugs. These thin and pliant insects are not heavily sclerotized and capable of squeezing under door thresholds or entering almost any opening to the home in their quest for a blood meal. The more people and pets living in the home, the greater the levels of the attractants, carbon dioxide (CO_2_) and lactic acid. Animals in the peridomestic space may also contribute to home entry where bugs move back and forth between hosts. For example, chickens are a favored host for blood meals, and coops adjacent to homes risk home entry by kissing bugs.

All homes are susceptible to intrusion. Whether old or new, using air conditioning or not, homes can and will be entered [7]. In the Sonoran Desert, this usually occurs in late May and June during the hours shortly after sunset as the desert floor warms [5]. Kissing bugs are poor fliers and need optimal conditions for flight during this time. They are strongly attracted to light and will land on well lighted walls, windows, screens and porches. We are aware of several people who have built new structures next to old permeable structures to escape kissing bugs in Arizona and Louisiana (personal observation of authors and P.L. Dorn). The principal advantage of the new structure in Arizona was that there was less clutter and, when bugs intruded, they were readily visible. Thus, building new structures will not keep the bugs out. This was also observed in Brazil where traditional housing was replaced with new tightly constructed units and the new homes became colonized with kissing bugs [12].

Kissing bug species found in the home are classified as intrusive, domiciliated or domestic. These categories are characterized by finding only adults and no eggs or nymphs for intrusive bugs; finding all life stages in the peridomestic or domestic environment for domiciliated bugs; and domesticated bugs are domiciliated, but found over an extensive geographic region [13]. Almost 20% of homes in Tucson with kissing bugs had nymphs present, which would be categorized as domiciliated. However, we are unaware of any homes that have year-round presence of nymphs and adults, which is the implication of the term domiciliated. Instead, intrusion becomes a yearly event with *T. rubida*, which does not demonstrate strong anthropophily.

Once bugs enter a home, the homeowner must find and destroy them. Years of experience with telephone calls and clinic visits have taught us that the most common site in the home to find these intruders is the bedroom. They are often between the mattress and box springs, and one must search diligently. Females entering the home in May will more than likely lay eggs, giving rise to nymphs. Thus, homeowners often submit an adult bug and 3–5 nymphs (examples 1 and 2 of emails). Application of a residual insecticide kills bugs, but the treatment needs to be applied on a yearly basis to be successful [10]. Another frequent site in the home where bugs can be found is behind bookshelves or furniture near where pets regularly sleep.

Not all kissing bug species are frequent intruders. Although seven species of kissing bugs are present in Arizona, complaints directed to us have mainly concerned *Triatoma rubida, T. protracta* and *T. recurva*. *Triatoma rubida* is the most common home intruder in the Sonoran Desert followed by *T. protracta*, which is infrequent [14]. *Triatoma recurva* has a different habitat than the other two species, usually confined to higher elevations such as Bisbee, AZ [7]. However, even in Bisbee, it is less common than *T. rubida* as a home intruder. No homeowner has sent us *Paratriatoma hirsuta*, which is found in the Mohave Desert or any other Arizonan species [14]. We have had many bugs sent to us from homes in San Diego, Los Angeles and Palm Springs, CA and all were *T. protracta*. Not surprisingly, the most populous species in an area will likely be the leading home intruder; in the Sonoran Desert, *T. rubida* is the most common home intruder.

Allergic reactions following a kissing bug bite are a problem but testing for Chagas disease is not warranted. Salivary allergens of kissing bugs are species-specific and range from trivial to life-threatening reactions [15]. Kissing bug bites are the most common cause of anaphylaxis from insect bites [16,17]. One individual in the Tucson home study experienced 11 episodes of anaphylaxis [7]. We studied *T. rubida, T. protracta* and *T. recurva* feeding habits on live rodents and in no instance did bugs defecate on the animals, on the contrary after feeding they attempted to move as far away as possible from the host and then only a minority defecated within 1 h after feeding [18]. This is presumably different than the behavior of *T. infestans*, which historically has been the vector of millions of human *T. cruzi* infections where percutaneous or conjunctival entry of the parasite is hypothesized. Although *T. rubida* is often in contact with humans in the Desert Southwest it does not seem to pose a great risk for Chagas disease at the time of feeding.

## 5. Conclusions

Recipients of kissing bug bites need not fear acquiring Chagas disease because the disease is not transmitted by bite saliva. We studied over 100 individuals who had been bitten in the aggregate, several thousand times, and no individual was infected with Chagas [5]. Since no single commercially available test serves as the gold standard diagnostic test, we do not recommend testing for this disease among victims of kissing bug bites.

In the future, with more people moving to the Southwest, the incidence of kissing bug home intrusions followed by biting incidents will become commonplace. Immunotherapy for anaphylaxis from kissing bug bites is desperately needed for some residents but, better education is needed for all Southwest residents and physicians about kissing bug behavior and their carriage of *T. cruzi*. Avoiding kissing bug bites is important in preventing severe allergies in the Southwest. The parasite carried by many kissing bugs, *T. cruzi*, is not found in the saliva, but in the rectum of kissing bugs and their feces; thus, residents should take care to avoid swallowing bugs or their feces that can contaminate food and water. In summary, preventing kissing bug entry into the home is an important skill for homeowners to acquire in order to live successfully alongside these ubiquitous bugs in the desert Southwest.

## Figures and Tables

**Table 1:** Results of Tucson homesites enrolled in the kissing bug study (78) and completed surveys (77).

**Home Characteristic**	**Numbers (%)**
Total homes studied	78
Homes with bugs captured inside	46 (58.9)
Homes with gravid female bugs	13 (16.7)
Homes with nymphs	9 (11.5)
Homes with bugs carrying *T. cruzi*	29 (37.2)
Invaded homes with bugs carrying *T. cruzi*	29 of 46 (63%)
**Pets and Livestock**	**N (%)**
Total surveys submitted	77
Homeowners with pets	53 (69)
Homes with pets living inside	46 (59.8)
Peridomestic space with chickens	10 (13)
Stray animal sited in peridomestic space in past month	15 (19.5)

## Data Availability

Data can be found in Supplementary item 1 and 2.

## Supplementary Materials

The following are available online at https://www.mdpi.com/article/10.3390/insects12070654/s1, Supplementary Data 1 and Supplementary Data 2.
