# Supplementary material for: Kissing Bug Intrusions into Homes in the Southwest United States
Source: Insects. 2021 Jul 17;12(7):654. doi: 10.3390/insects12070654 (PMC8306929; doi:10.3390/insects12070654)
Supplement: Supplementary file 1 [file insects-12-00654-s001.zip › insects-1278075-Supplement1.pdf]

University of Arizona
Center for Biomedical Informatics & Biostatistics

Tucson Kissing bug Project Home and Personal Evaluation

Data Exports, Reports, and Stats

All data (all records and fields)

Data entry volunteer: (Please put first name, initial of last name) Example: Norman Beatty: NormanB *(data_entery_vol)*

Total Count (N)	Missing
77	1 (1.3%)

Household Identification Number: (Survey ID) *(house_id)*

Total Count (N)	Missing
78	0 (0.0%)

Date of survey complete: (MM/DD/YY) *(date_survey)*

Total Count (N)	Missing
77	1 (1.3%)

How long have you lived in your home? (years) *(home_how_long_years)*

Total Count (N)	Missing	Unique
72	6 (7.7%)	33

Counts/frequency: < 1 (1, 1.4%), 1 (3, 4.2%), 2 (6, 8.3%), 3 (4, 5.6%), 4 (1, 1.4%), 5 (4, 5.6%), 6 (3, 4.2%), 7 (1, 1.4%), 8 (2, 2.8%), 9 (2, 2.8%), 10 (3, 4.2%), 11 (3, 4.2%), 12 (2, 2.8%), 13 (5, 6.9%), 14 (3, 4.2%), 15 (1, 1.4%), 16 (4, 5.6%), 17 (0, 0.0%), 18 (3, 4.2%), 19 (1, 1.4%), 20 (4, 5.6%), 21 (1, 1.4%), 22 (1, 1.4%), 23 (0, 0.0%), 24 (2, 2.8%), 25 (0, 0.0%), 26 (0, 0.0%), 27 (0, 0.0%), 28 (1, 1.4%), 29 (2, 2.8%), 30 (0, 0.0%), 31 (0, 0.0%), 32 (0, 0.0%), 33 (1, 1.4%), 34 (1, 1.4%), 35 (1, 1.4%), 36 (0, 0.0%), 37 (1, 1.4%), 38 (0, 0.0%), 39 (0, 0.0%), 40 (2, 2.8%), 41 (0, 0.0%), 42 (1, 1.4%), 43 (0, 0.0%), 44 (0, 0.0%), 45 (0, 0.0%), 46 (0, 0.0%), 47 (1, 1.4%), 48 (1, 1.4%), 49 (0, 0.0%), 50 (0, 0.0%), 51 (0, 0.0%), 52 (0, 0.0%), 53 (0, 0.0%), 54 (0, 0.0%), 55 (0, 0.0%), 56 (0, 0.0%), 57 (0, 0.0%), 58 (0, 0.0%), 59 (0, 0.0%), 60 (0, 0.0%), 61 (0, 0.0%), 62 (0, 0.0%), 63 (0, 0.0%), 64 (0, 0.0%), 65 (0, 0.0%), 66 (0, 0.0%), 67 (0, 0.0%), 68 (0, 0.0%), 69 (0, 0.0%), 70 (0, 0.0%), 71 (0, 0.0%), 72 (0, 0.0%), 73 (0, 0.0%), 74 (0, 0.0%), 75 (0, 0.0%), 76 (0, 0.0%), 77 (0, 0.0%), 78 (0, 0.0%), 79 (0, 0.0%), 80 (0, 0.0%), >80 (0, 0.0%)

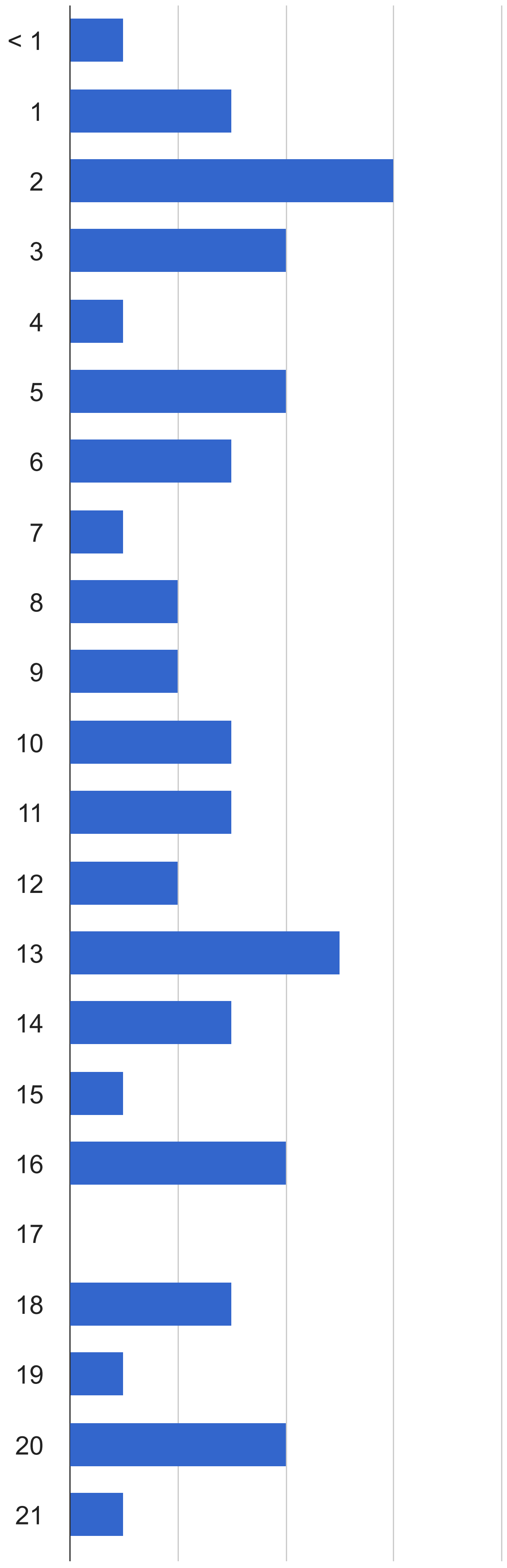
< 1
1
2
3
4
5
6
7
8
9
10
11
12
13
14
15
16
17
18
19
20
21

Download image

Do you own or rent? *(home_or_rent)*

Total Count (N)	Missing	Unique
74	4 (5.1%)	2

Counts/frequency: Own (65, 87.8%), Rent (9, 12.2%)

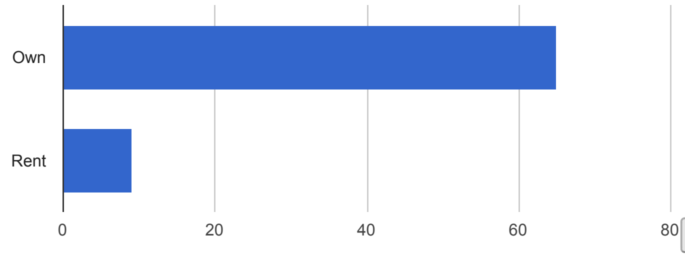

Download image

Approximately how old is your home? (years) *(home_how_old_yrs)*

Total Count (N)	Missing	Unique
76	2 (2.6%)	43

Counts/frequency: < 1 (0, 0.0%), 1 (0, 0.0%), 2 (2, 2.6%), 3 (0, 0.0%), 4 (0, 0.0%), 5 (0, 0.0%), 6 (1, 1.3%), 7 (1, 1.3%), 8 (0, 0.0%), 9 (1, 1.3%), 10 (1, 1.3%), 11 (1, 1.3%), 12 (2, 2.6%), 13 (0, 0.0%), 14 (2, 2.6%), 15 (3, 3.9%), 16 (2, 2.6%), 17 (1, 1.3%), 18 (1, 1.3%), 19 (1, 1.3%), 20 (5, 6.6%), 21 (0, 0.0%), 22 (0, 0.0%), 23 (0, 0.0%), 24 (2, 2.6%), 25 (3, 3.9%), 26 (2, 2.6%), 27 (0, 0.0%), 28 (0, 0.0%), 29 (1, 1.3%), 30 (2, 2.6%), 31 (0, 0.0%), 32 (1, 1.3%), 33 (1, 1.3%), 34 (1, 1.3%), 35 (6, 7.9%), 36 (1, 1.3%), 37 (1, 1.3%), 38 (1, 1.3%), 39 (0, 0.0%), 40 (8, 10.5%), 41 (1, 1.3%), 42 (1, 1.3%), 43 (0, 0.0%), 44 (0, 0.0%), 45 (3, 3.9%), 46 (0, 0.0%), 47 (1, 1.3%), 48 (1, 1.3%), 49 (0, 0.0%), 50 (1, 1.3%), 51 (1, 1.3%), 52 (0, 0.0%), 53 (0, 0.0%), 54 (2, 2.6%), 55 (0, 0.0%), 56 (1, 1.3%), 57 (1, 1.3%), 58 (0, 0.0%), 59 (0, 0.0%), 60 (3, 3.9%), 61 (0, 0.0%), 62 (0, 0.0%), 63 (0, 0.0%), 64 (0, 0.0%), 65 (1, 1.3%), 66 (1, 1.3%), 67 (0, 0.0%), 68 (0, 0.0%), 69 (0, 0.0%), 70 (2, 2.6%), 71 (0, 0.0%), 72 (0, 0.0%), 73 (0, 0.0%), 74 (0, 0.0%), 75 (0, 0.0%), 76 (0, 0.0%), 77 (0, 0.0%), 78 (0, 0.0%), 79 (0, 0.0%), 80 (0, 0.0%), 81 (0, 0.0%), 82 (0, 0.0%), 83 (0, 0.0%), 84 (0, 0.0%), 85 (0, 0.0%), 86 (0, 0.0%), 87 (0, 0.0%), 88 (1, 1.3%), 89 (0, 0.0%), 90 (0, 0.0%), 91 (0, 0.0%), 92 (0, 0.0%), 93 (0, 0.0%), 94 (1, 1.3%), 95 (0, 0.0%), 96 (0, 0.0%), 97 (0, 0.0%), 98 (0, 0.0%), 99 (0, 0.0%), 100 (0, 0.0%), >100 (0, 0.0%)

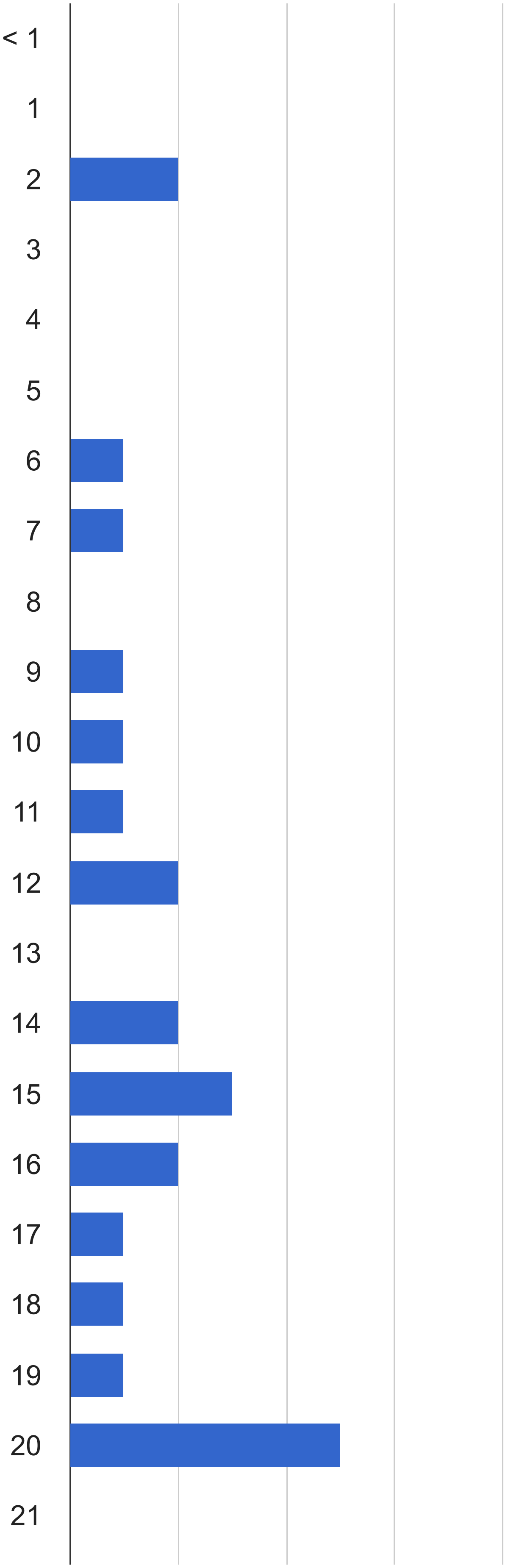

< 1
1
2
3
4
5
6
7
8
9
10
11
12
13
14
15
16
17
18
19
20
21

Download image

How many people live in the home? *(home_how_many_live)*

Total Count (N)	Missing	Unique
77	1 (1.3%)	6

Counts/frequency: 1 (18, 23.4%), 2 (43, 55.8%), 3 (8, 10.4%), 4 (6, 7.8%), 5 (1, 1.3%), 6 (1, 1.3%), 7 (0, 0.0%), 8 (0, 0.0%), 9 (0, 0.0%), 10 (0, 0.0%), 11 (0, 0.0%), 12 (0, 0.0%), 13 (0, 0.0%), 14 (0, 0.0%), 15 (0, 0.0%), >15 (0, 0.0%)

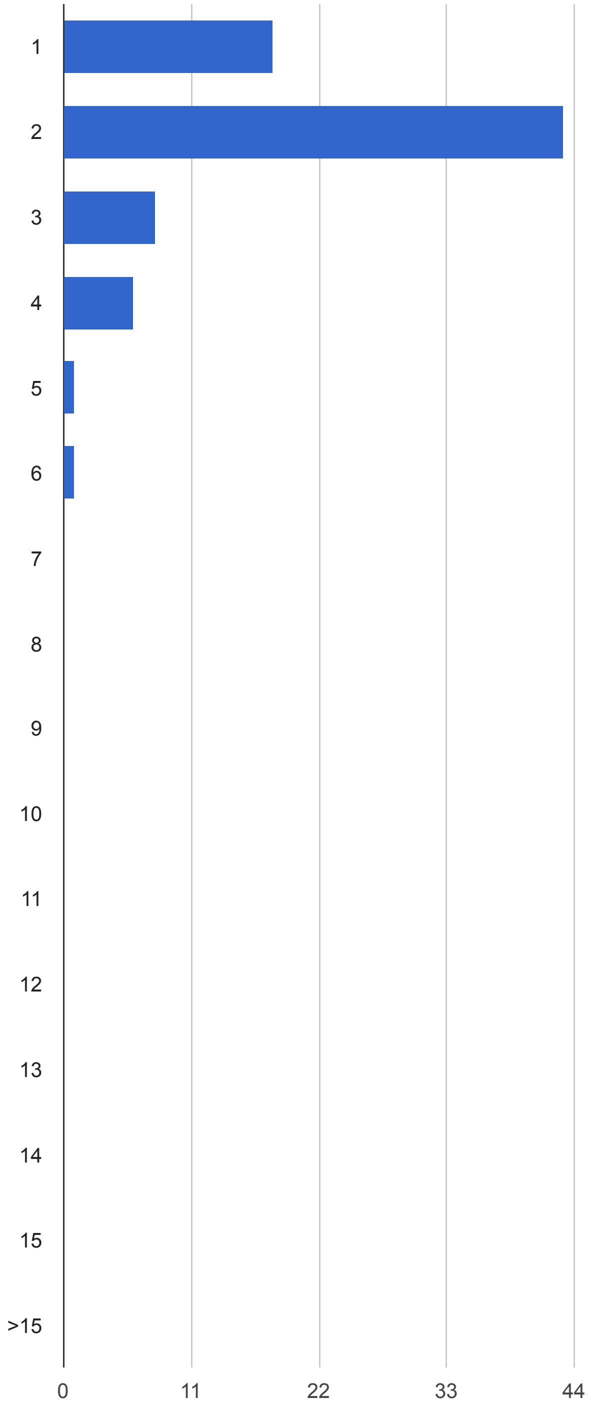

Download image

Do you have any pets? *(pets_y_n)*

Total Count (N)	Missing	Unique
77	1 (1.3%)	2

Counts/frequency: Yes (53, 68.8%), No (24, 31.2%)

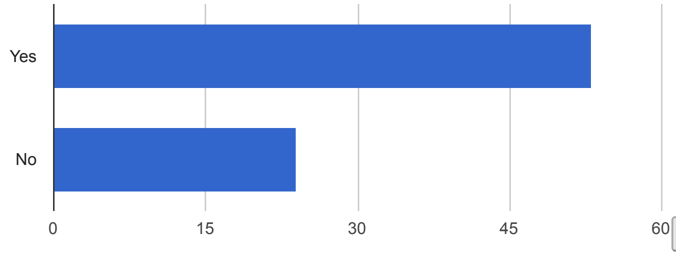

Download image

Which type of pet? *(type_pet)*

Total Count (N)	Missing	Unique
53	25 (32.1%)	3

Counts/frequency: Dog (40, 75.5%), Cat (29, 54.7%), Other (14, 26.4%)

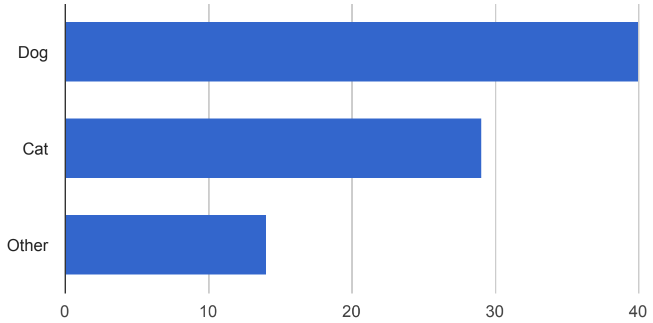

Download image

How many dogs? *(dogs_quantity)*

Total Count (N)	Missing	Unique
40	38 (48.7%)	6

Counts/frequency: 1 (20, 50.0%), 2 (12, 30.0%), 3 (2, 5.0%), 4 (4, 10.0%), 5 (0, 0.0%), 6 (1, 2.5%), 7 (0, 0.0%), 8 (1, 2.5%), 9 (0, 0.0%), 10 (0, 0.0%), >10 (0, 0.0%)

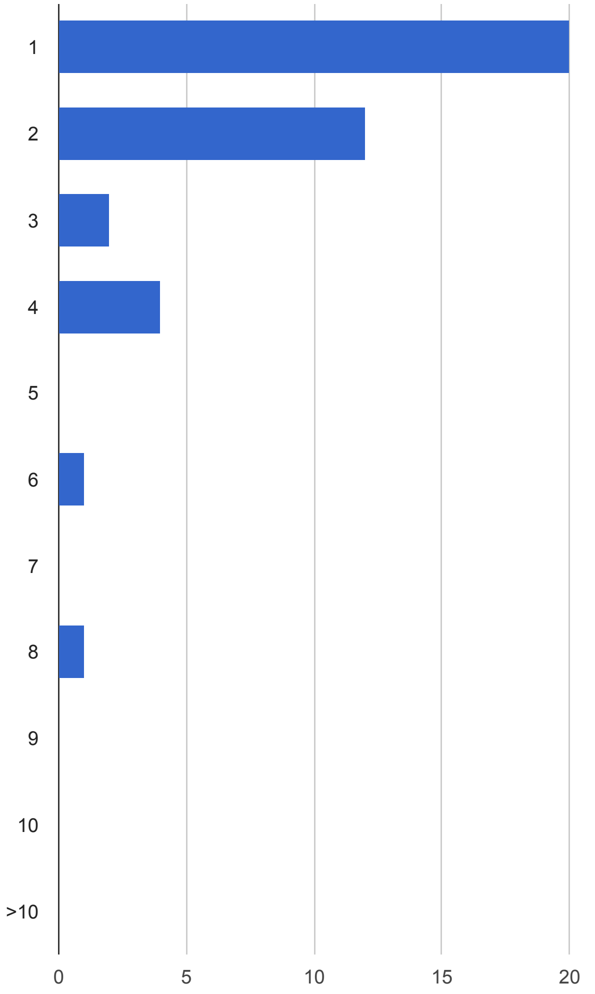

Download image

How many cats? *(cats_quantity)*

Total Count (N)	Missing	Unique
29	49 (62.8%)	6

Counts/frequency: 1 (9, 31.0%), 2 (11, 37.9%), 3 (5, 17.2%), 4 (1, 3.4%), 5 (2, 6.9%), 6 (1, 3.4%), 7 (0, 0.0%), 8 (0, 0.0%), 9 (0, 0.0%), 10 (0, 0.0%), 11 (0, 0.0%), 12 (0, 0.0%), 13 (0, 0.0%), 14 (0, 0.0%), 15 (0, 0.0%), >16 (0, 0.0%)

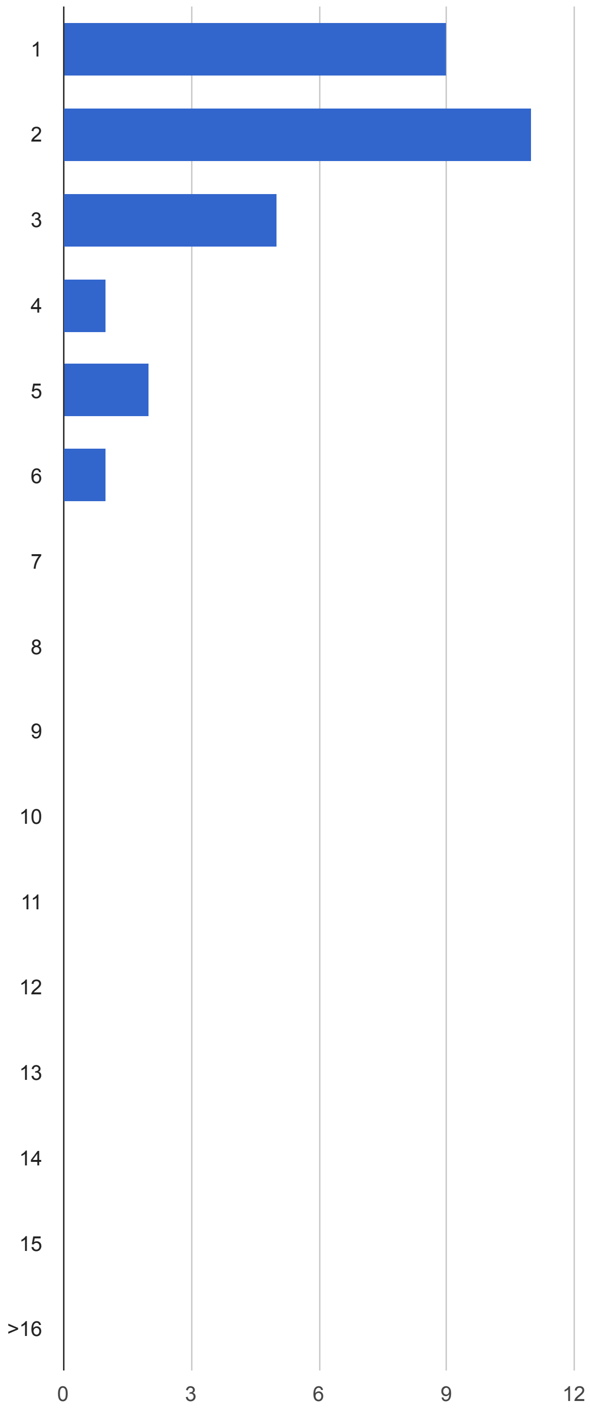

Download image

Do the dogs sleep inside or outside? *(dogs_inside_outside)*

Total Count (N)	Missing	Unique
40	38 (48.7%)	2

Counts/frequency: Inside (38, 95.0%), Outside (2, 5.0%)

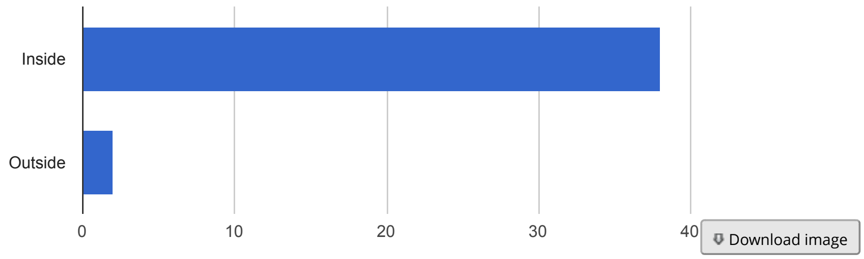

Dogs Notes: *(dogs_notes)*

Total Count (N)	Missing
8	70 (89.7%)

Do the cats sleep inside or outside? *(cats_inside_outside)*

Total Count (N)	Missing	Unique
29	49 (62.8%)	2

Counts/frequency: Inside (26, 89.7%), Outside (3, 10.3%)

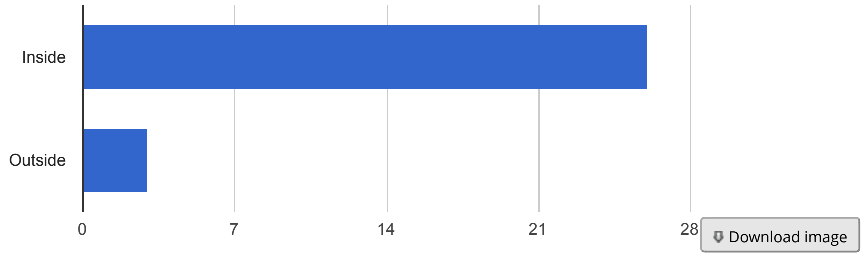

Cats Notes: *(cats_notes)*

Total Count (N)	Missing
11	67 (85.9%)

What "other" pets do you have? *(other_pets)*

Total Count (N)	Missing
14	64 (82.1%)

Do the "other" pets sleep inside or outside? *(other_pet_inside_outside)*

Total Count (N)	Missing	Unique
14	64 (82.1%)	2

Counts/frequency: Inside (8, 57.1%), Outside (6, 42.9%)

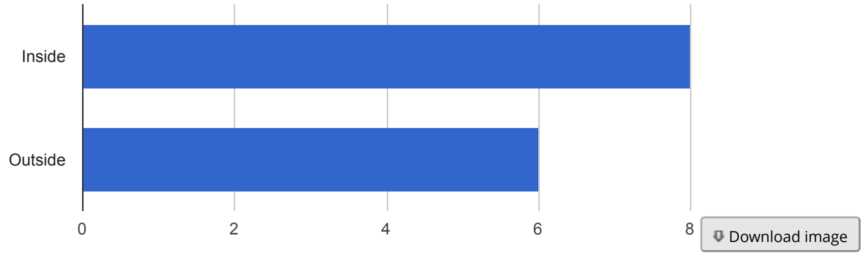

"Other" Pets Notes: *(other_pets_notes)*

Total Count (N)	Missing
1	77 (98.7%)

Do you have any livestock? *(livestock_y_n)*

Total Count (N)	Missing	Unique
77	1 (1.3%)	2

Counts/frequency: Yes (17, 22.1%), No (60, 77.9%)

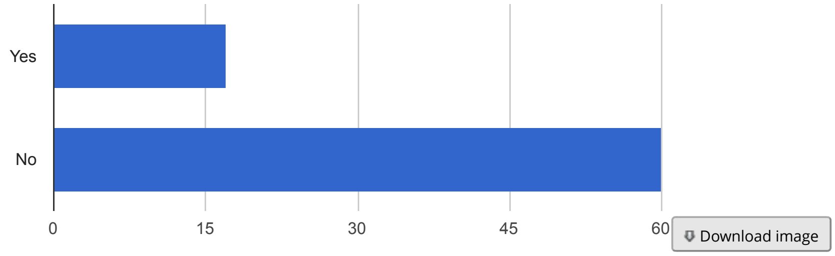

What type of livestock do you have? *(livestock_type)*

Total Count (N)	Missing	Unique
17	61 (78.2%)	5

Counts/frequency: Cattle (3, 17.6%), Horses (9, 52.9%), Goats/Sheep (2, 11.8%), Chickens (7, 41.2%), Pigs (0, 0.0%), Turkey (0, 0.0%), Llama (0, 0.0%), Other (1, 5.9%)

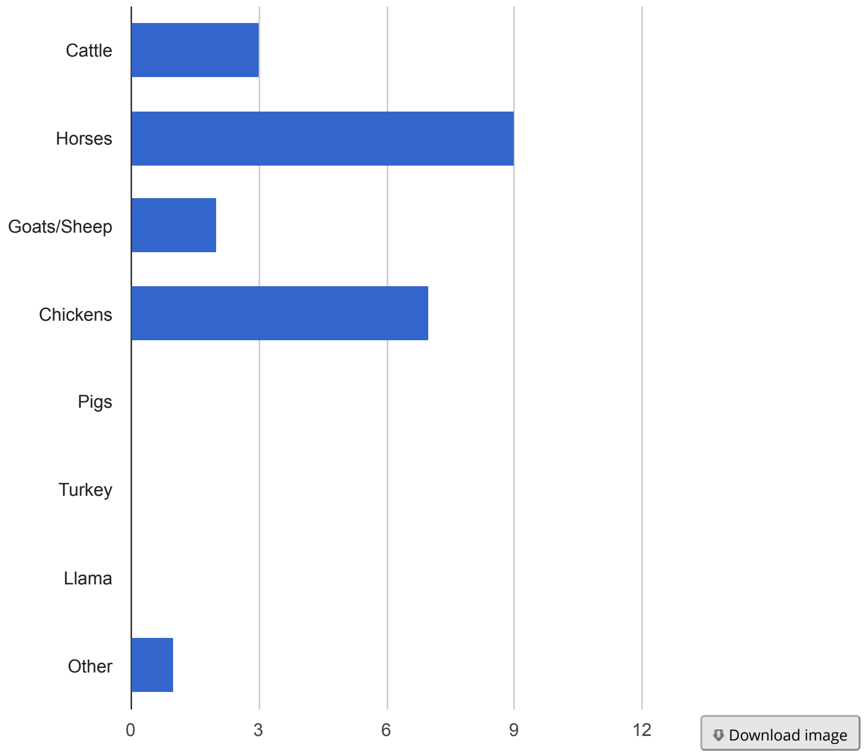

How many cattle? *(cattle_quantity)*

Total Count (N)	Missing	Unique
3	75 (96.2%)	1

Counts/frequency: 1 (0, 0.0%), 2 (0, 0.0%), 3 (0, 0.0%), 4 (0, 0.0%), 5 (0, 0.0%), 6 (0, 0.0%), 7 (0, 0.0%), 8 (0, 0.0%), 9 (0, 0.0%), 10 (0, 0.0%), 11 (0, 0.0%), 12 (0, 0.0%), 13 (0, 0.0%), 14 (0, 0.0%), 15 (0, 0.0%), >15 (3, 100.0%)

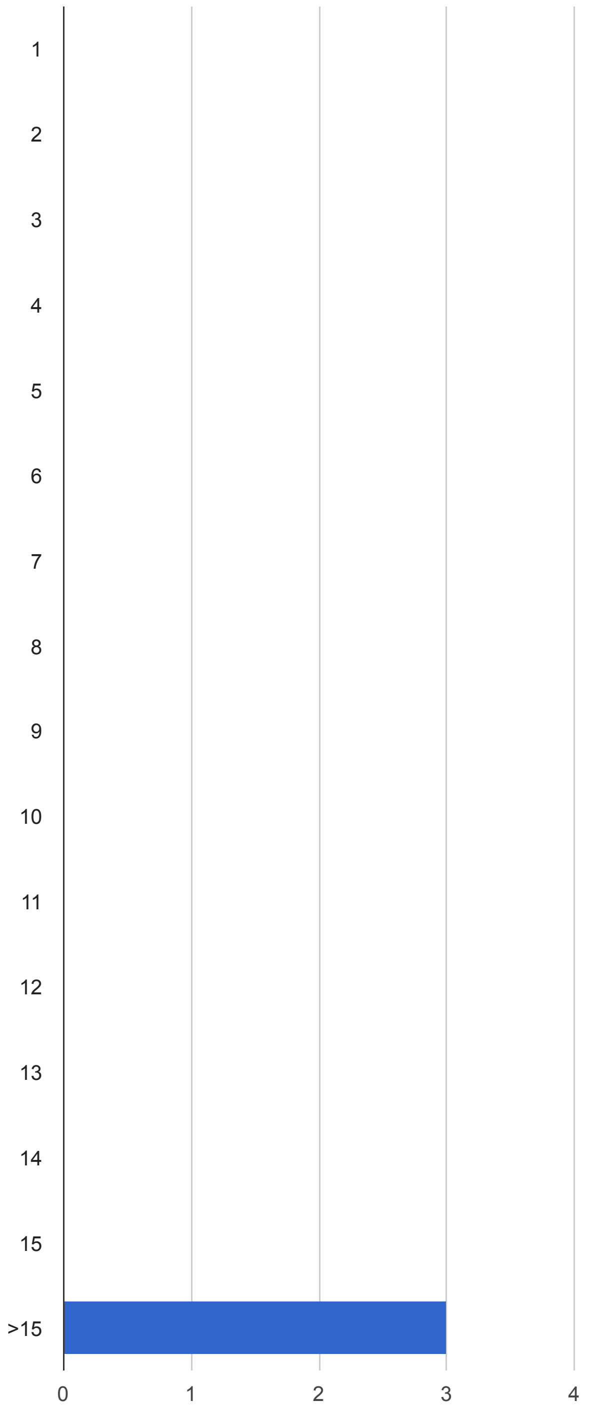

Download image

How many Horses? *(horses_quantity)*

Total Count (N)	Missing	Unique
8	70 (89.7%)	5

Counts/frequency: 1 (1, 12.5%), 2 (4, 50.0%), 3 (0, 0.0%), 4 (1, 12.5%), 5 (0, 0.0%), 6 (0, 0.0%), 7 (0, 0.0%), 8 (0, 0.0%), 9 (0, 0.0%), 10 (0, 0.0%), 11 (0, 0.0%), 12 (0, 0.0%), 13 (0, 0.0%), 14 (0, 0.0%), 15 (1, 12.5%), >15 (1, 12.5%)

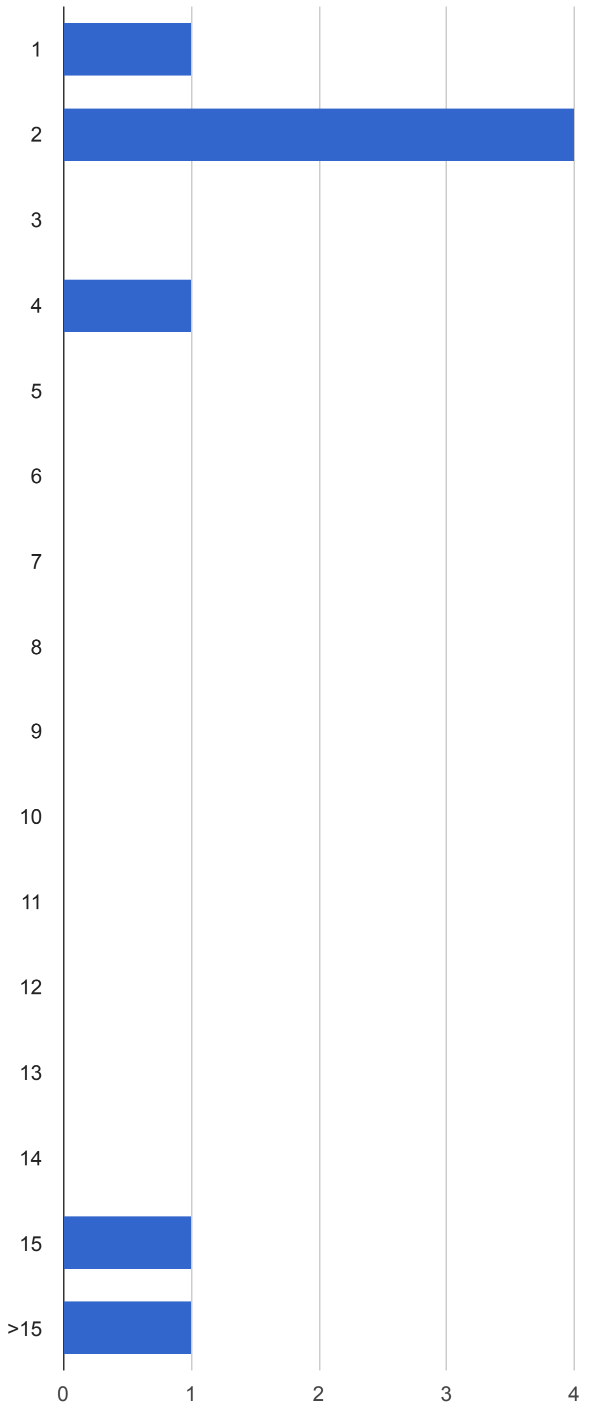

Download image

How many goats/sheep? *(goats_sheep_quantity)*

Total Count (N)	Missing	Unique
2	76 (97.4%)	2

Counts/frequency: 1 (0, 0.0%), 2 (1, 50.0%), 3 (0, 0.0%), 4 (0, 0.0%), 5 (1, 50.0%), 6 (0, 0.0%), 7 (0, 0.0%), 8 (0, 0.0%), 9 (0, 0.0%), 10 (0, 0.0%), 11 (0, 0.0%), 12 (0, 0.0%), 13 (0, 0.0%), 14 (0, 0.0%), 15 (0, 0.0%), >15 (0, 0.0%)

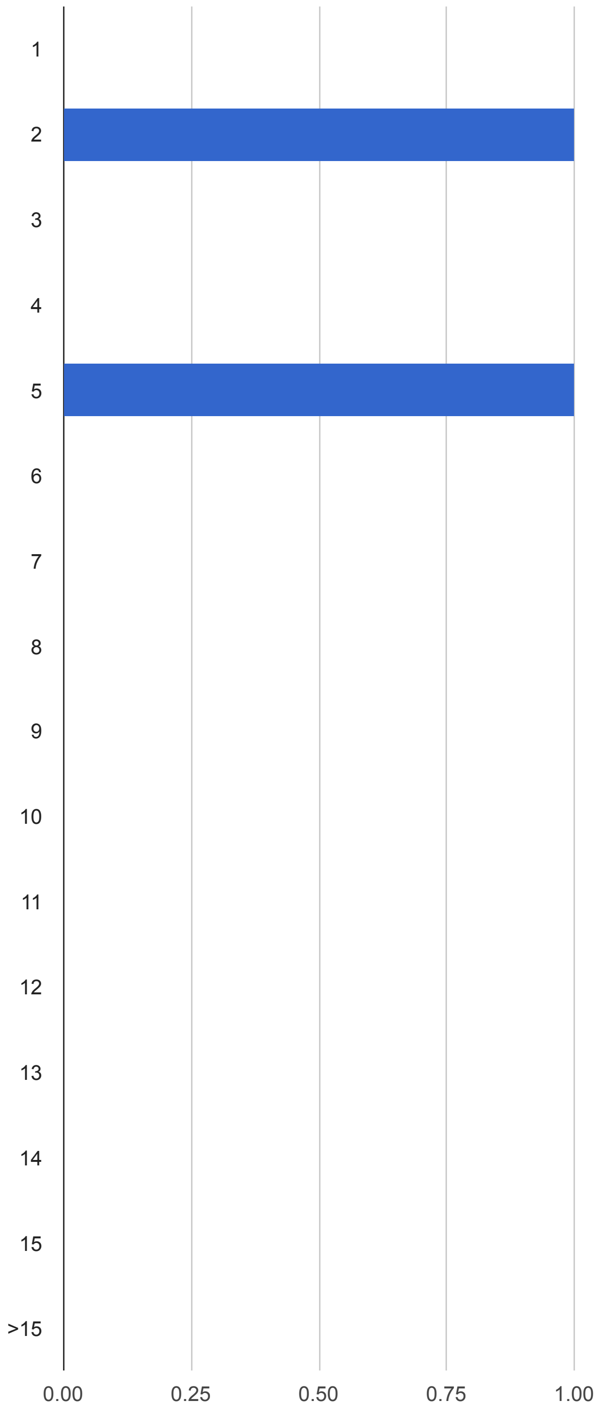

Download image

How many chickens? *(chickens_quantity)*

Total Count (N)	Missing	Unique
6	72 (92.3%)	5

Counts/frequency: 1 (0, 0.0%), 2 (2, 33.3%), 3 (1, 16.7%), 4 (1, 16.7%), 5 (0, 0.0%), 6 (1, 16.7%), 7 (0, 0.0%), 8 (0, 0.0%), 9 (0, 0.0%), 10 (1, 16.7%), 11 (0, 0.0%), 12 (0, 0.0%), 13 (0, 0.0%), 14 (0, 0.0%), 15 (0, 0.0%), >15 (0, 0.0%)

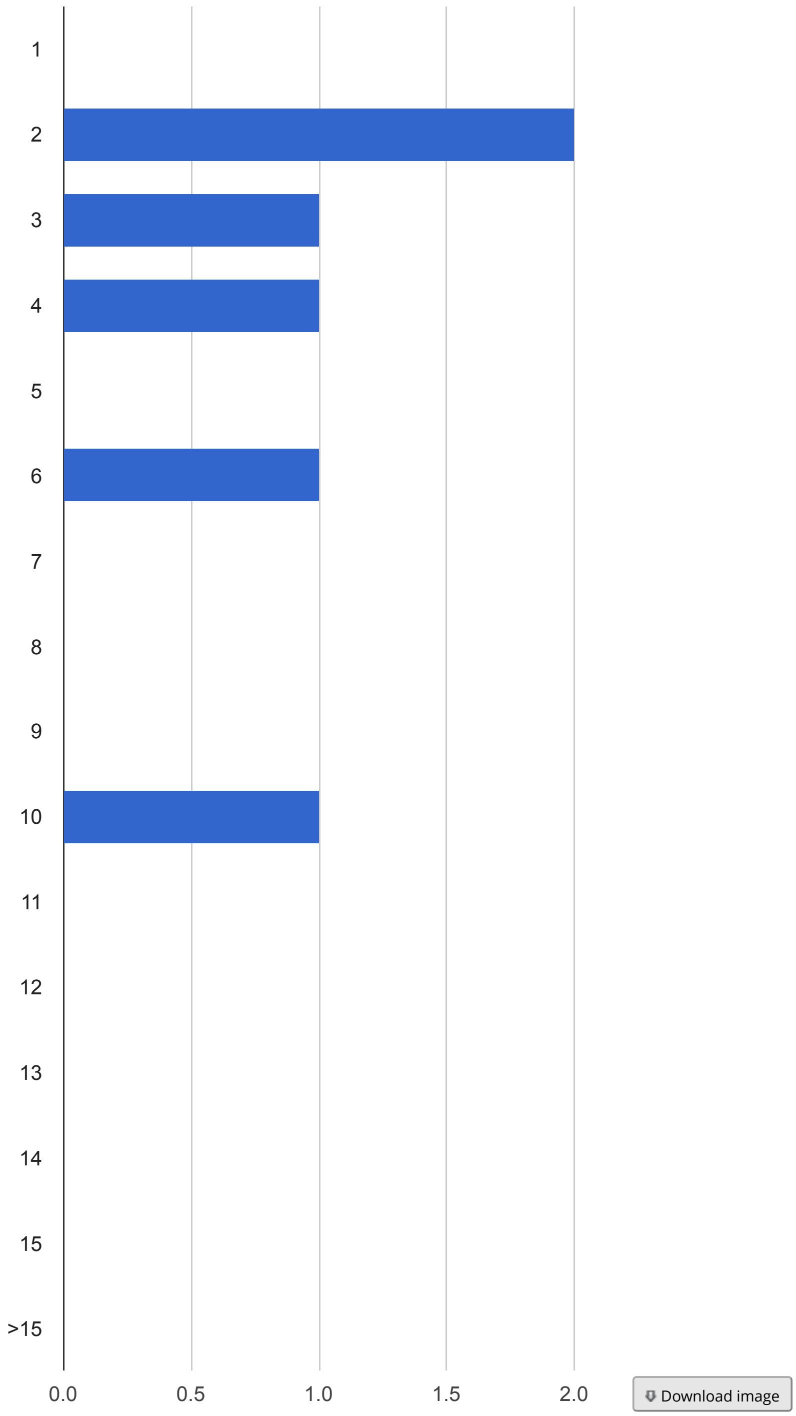

How many pigs? *(pigs_quantity)*

Total Count (N)	Missing
0	78 (100.0%)

How many turkeys? *(turkey_quantity)*

Total Count (N)	Missing

0	78 (100.0%)

How many llamas? *(llama_quantity)*

Total Count (N)	Missing
0	78 (100.0%)

What "other" livestock? *(livestock_other)*

Total Count (N)	Missing
1	77 (98.7%)

How many of this "other" livestock? *(livestock_other_quant)*

Total Count (N)	Missing
0	78 (100.0%)

Within the last month have you seen wild, stray animals, or rodents in your house, attic or under your house? *(wild_stray_rodents_y_n)*

Total Count (N)	Missing	Unique
77	1 (1.3%)	2

Counts/frequency: Yes (21, 27.3%), No (56, 72.7%)

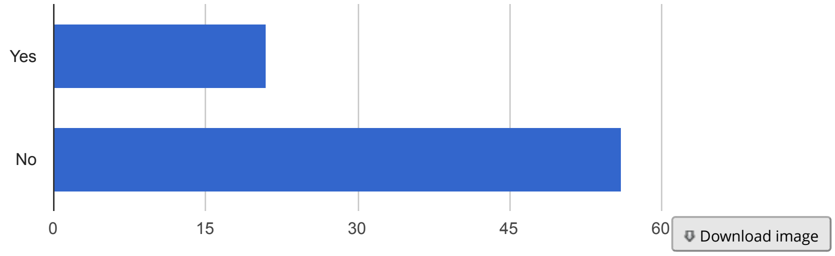

Do you have air conditioning? *(air_conditioning_y_n)*

Total Count (N)	Missing	Unique
77	1 (1.3%)	2

Counts/frequency: Yes (64, 83.1%), No (13, 16.9%)

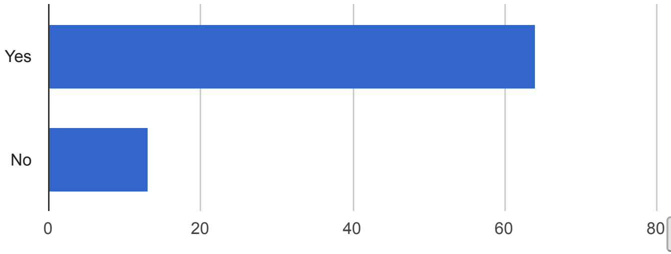

Download image

During the last month, how often did you use the air-conditioning at night? *(air_conditioning_how_often)*

Total Count (N)	Missing	Unique
69	9 (11.5%)	3

Counts/frequency: 4 or more nights/week (52, 75.4%), 3 or fewer nights/week (4, 5.8%), Never (13, 18.8%)

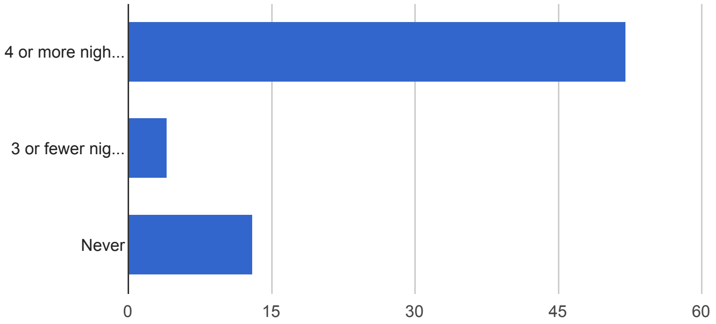

Download image

Air conditioning: Additional Notes *(air_conditioning_notes)*

Total Count (N)	Missing
22	56 (71.8%)

Do you have screen on your windows? *(screens_windows_y_n)*

Total Count (N)	Missing	Unique
77	1 (1.3%)	2

Counts/frequency: Yes (75, 97.4%), No (2, 2.6%)

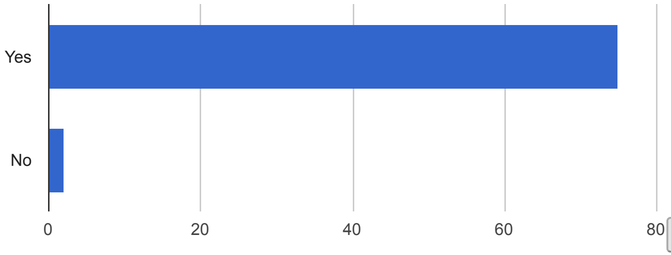

Download image

During the last month, how often do you leave windows open? *(windows_open_how_often)*

Total Count (N)	Missing	Unique
76	2 (2.6%)	3

Counts/frequency: 4 or more nights/week (30, 39.5%), 3 or fewer nights/week (10, 13.2%), Never (36, 47.4%)

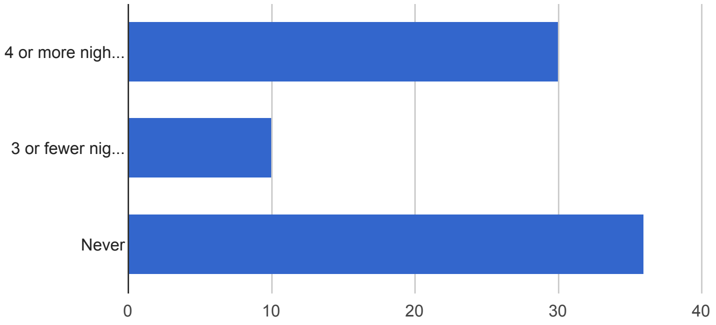

Download image

Doors/Windows screens: Additional notes *(screens_notes)*

Total Count (N)	Missing
18	60 (76.9%)

Do you apply any pesticides INSIDE your home? *(pesticides_inside_y_n)*

Total Count (N)	Missing	Unique
77	1 (1.3%)	2

Counts/frequency: Yes (26, 33.8%), No (51, 66.2%)

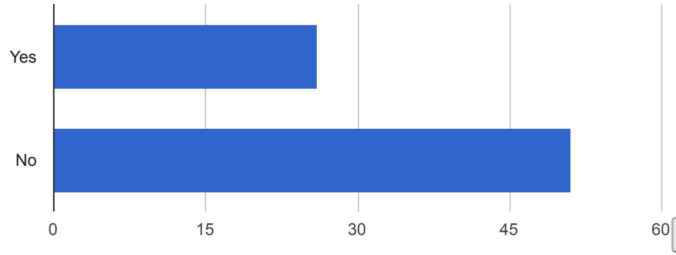

Download image

Do you apply any pesticides OUTSIDE your home? *(pesticides_outside_y_n)*

Total Count (N)	Missing	Unique
77	1 (1.3%)	2

Counts/frequency: Yes (33, 42.9%), No (44, 57.1%)

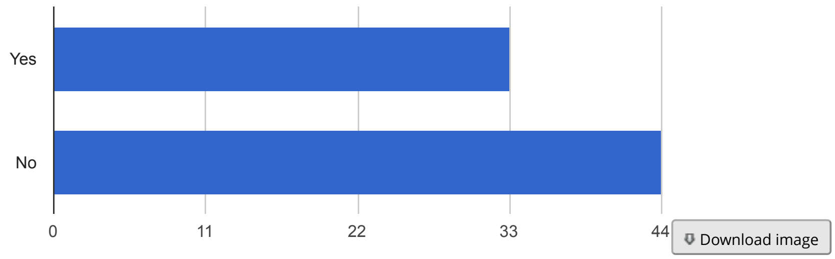

Pesticides: Additional notes *(pesticides_notes)*

Total Count (N)	Missing
32	46 (59.0%)

Do you have a professional pest control service? *(pest_control_y_n)*

Total Count (N)	Missing	Unique
77	1 (1.3%)	2

Counts/frequency: Yes (15, 19.5%), No (62, 80.5%)

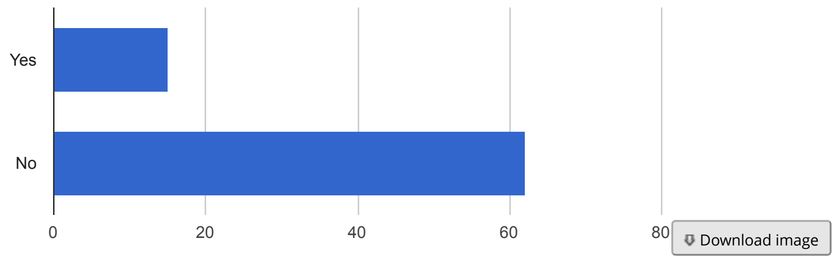

Do they spray inside, outside, or both? *(pest_control_location)*

Total Count (N)	Missing	Unique
16	62 (79.5%)	2

Counts/frequency: Inside (0, 0.0%), Outside (5, 31.3%), Both (11, 68.8%)

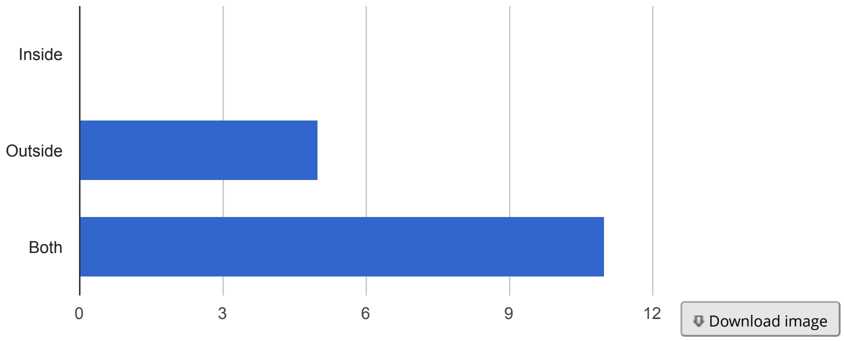

What is the name of the pest control company? *(pest_control_company)*

Total Count (N)	Missing
15	63 (80.8%)

Have you seen this insect? (participant acknowledges seeing a kissing bug before) *(seen_kissing_bug_y_n)*

Total Count (N)	Missing	Unique
77	1 (1.3%)	2

Counts/frequency: Yes (74, 96.1%), No (3, 3.9%)

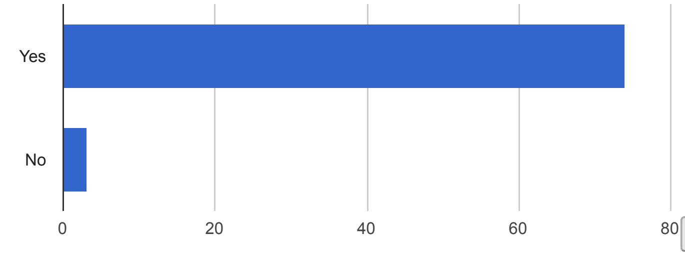

Download image

Where have you seen this insect (kissing bug)? *(seen_kissing_bug_location)*

Total Count (N)	Missing	Unique
75	3 (3.8%)	3

Counts/frequency: Inside (32, 42.7%), Outside (1, 1.3%), Both (42, 56.0%)

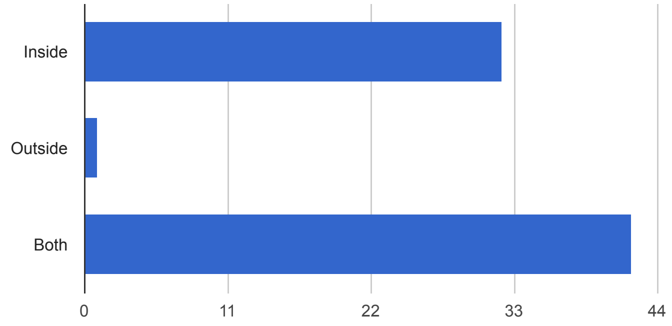

Download image

If inside your house, where have you seen it? *(inside_where)*

Total Count (N)	Missing
69	9 (11.5%)

If outside your house, where have you seen it? *(outside_where)*

Total Count (N)	Missing

57	21 (26.9%)

When did you last see this bug? (if participant only put a "date" please estimate approximately how many days or weeks or years from day survey was completed) *(last_seen_kissing_bug)*

Total Count (N)	Missing
76	2 (2.6%)

Is this house a primary house or secondary structure? *(home_primary_secondary)*

Total Count (N)	Missing	Unique
76	2 (2.6%)	2

Counts/frequency: Primary (75, 98.7%), Secondary (1, 1.3%)

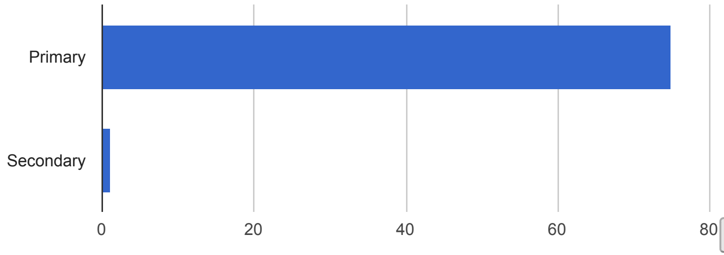

Download image

Is this house: (check all that apply) *(home_building_materials)*

Total Count (N)	Missing	Unique
75	3 (3.8%)	4

Counts/frequency: Raised (14, 18.7%), Concrete foundation (62, 82.7%), Brick (24, 32.0%), Siding (5, 6.7%)

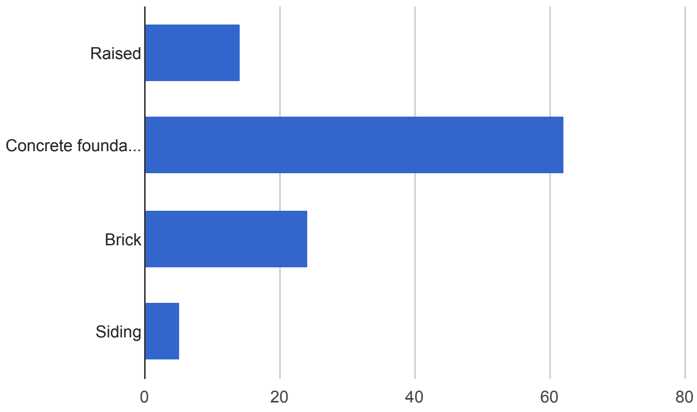

Download image

Assess structural integrity of Residence: 3 (good or great condition) 2 (decent condition) 1 (poor condition) *(home_condition_1_3)*

Total Count (N)	Missing	Unique
74	4 (5.1%)	3

Counts/frequency: 3 (48, 64.9%), 2 (24, 32.4%), 1 (2, 2.7%)

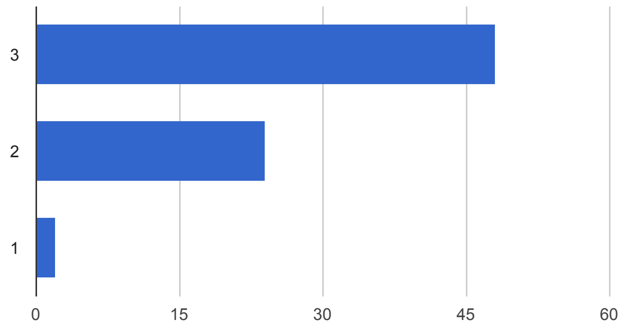

Download image

What type of material is the roof made of? *(home_roof_material)*

Total Count (N)	Missing	Unique
76	2 (2.6%)	4

Counts/frequency: Creosote tile/shingle (13, 17.1%), Ceramic tile (9, 11.8%), Metal (19, 25.0%), Other (40, 52.6%)

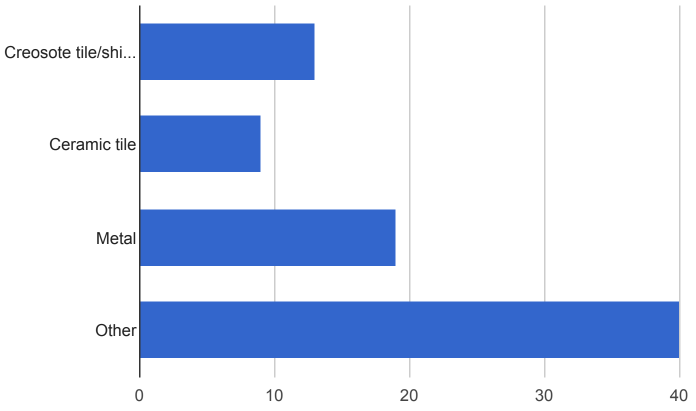

Download image

What "other' type of roofing material? *(home_roof_other)*

Total Count (N)	Missing
37	41 (52.6%)

From the perimeter of the house, does there appear to be holes or burrows under the house? *(burrows_house_y_n)*

Total Count (N)	Missing	Unique
77	1 (1.3%)	2

Counts/frequency: Yes (26, 33.8%), No (51, 66.2%)

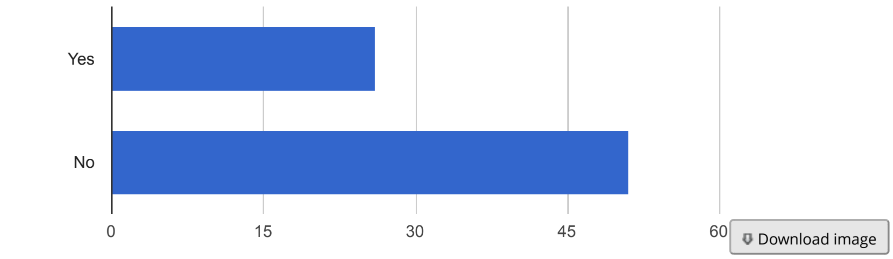

Does the home have screens on its doors? *(home_doors_screens_y_n)*

Total Count (N)	Missing	Unique
76	2 (2.6%)	2

Counts/frequency: Yes (47, 61.8%), No (29, 38.2%)

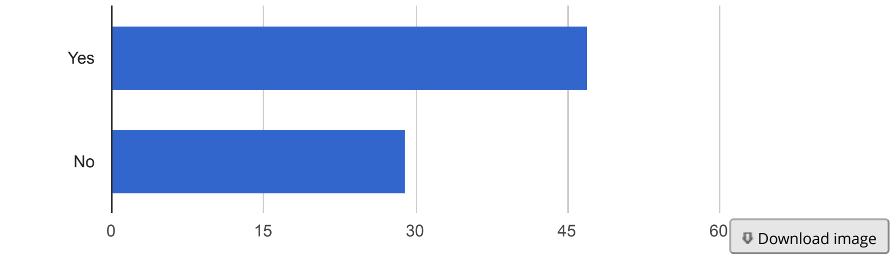

if "yes", the present condition of the door screens appear to be: (check only one) *(home_doors_screens_cond)*

Total Count (N)	Missing	Unique
46	32 (41.0%)	3

Counts/frequency: All screens intact (no holes) (34, 73.9%), Some screens have holes (11, 23.9%), Many screens have holes (1, 2.2%)

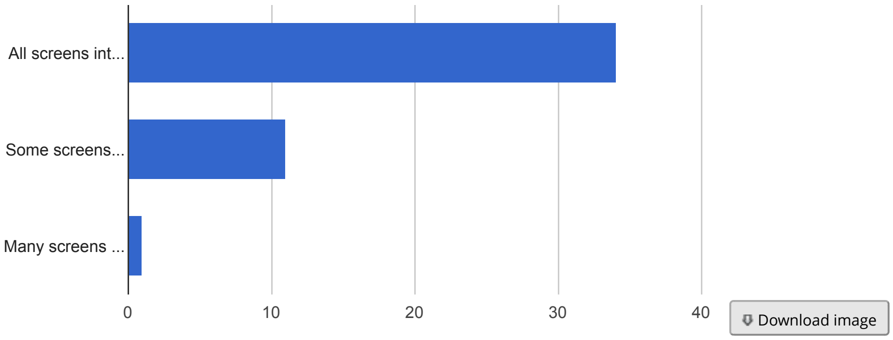

How many doors have screens? *(home_doors_screens_quant_y)*

Total Count (N)	Missing	Unique
68	10 (12.8%)	9

Counts/frequency: 0 (21, 30.9%), 1 (11, 16.2%), 2 (19, 27.9%), 3 (8, 11.8%), 4 (3, 4.4%), 5 (2, 2.9%), 6 (0, 0.0%), 7 (1, 1.5%), 8 (2, 2.9%), 9 (0, 0.0%), 10 (0, 0.0%), 11 (0, 0.0%), 12 (0, 0.0%), 13 (1, 1.5%), 14 (0, 0.0%), 15 (0, 0.0%), >15 (0, 0.0%)

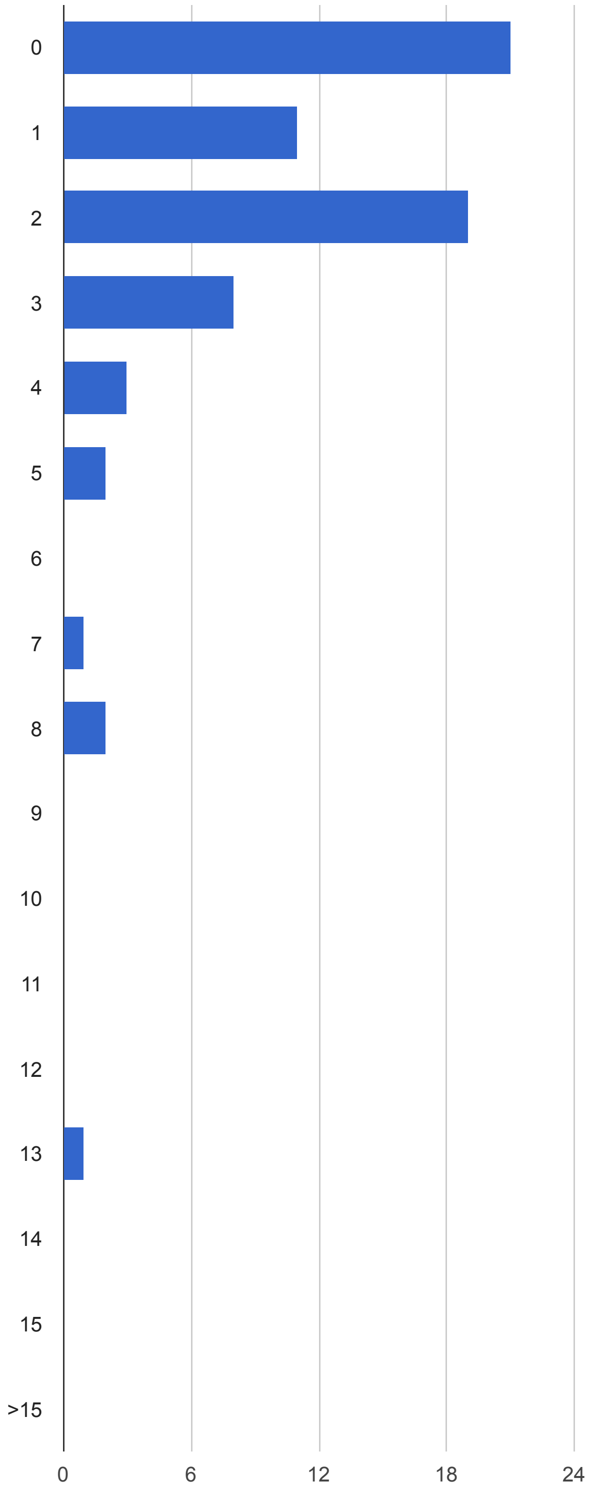

Download image

How many doors do not have screens? *(home_doors_screen_n)*

Total Count (N)	Missing	Unique
60	18 (23.1%)	8

Counts/frequency: 0 (18, 30.0%), 1 (9, 15.0%), 2 (17, 28.3%), 3 (7, 11.7%), 4 (5, 8.3%), 5 (2, 3.3%), 6 (0, 0.0%), 7 (1, 1.7%), 8 (0, 0.0%), 9 (1, 1.7%), 10 (0, 0.0%), 11 (0, 0.0%), 12 (0, 0.0%), 13 (0, 0.0%), 14 (0, 0.0%), 15 (0, 0.0%), >15 (0, 0.0%)

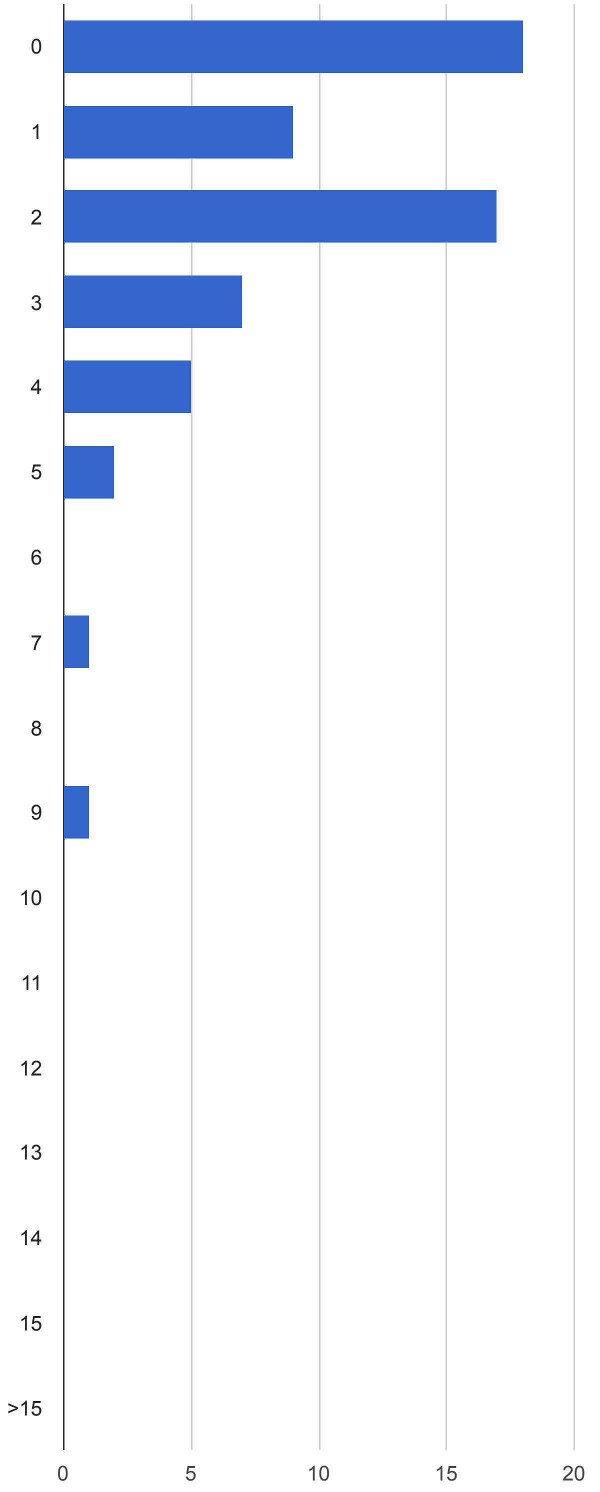

Download image

Household Attributes Additional Notes: *(home_additional_notes)*

Total Count (N)	Missing
16	62 (79.5%)

What number of outbuildings are on the property? *(outbuildings_quantity)*

Total Count (N)	Missing	Unique
76	2 (2.6%)	9

Counts/frequency: 0 (18, 23.7%), 1 (28, 36.8%), 2 (13, 17.1%), 3 (8, 10.5%), 4 (3, 3.9%), 5 (3, 3.9%), 6 (1, 1.3%), 7 (0, 0.0%), 8 (1, 1.3%), 9 (1, 1.3%), 10 (0, 0.0%), 11 (0, 0.0%), 12 (0, 0.0%), 13 (0, 0.0%), 14 (0, 0.0%), 15 (0, 0.0%), >15 (0, 0.0%)

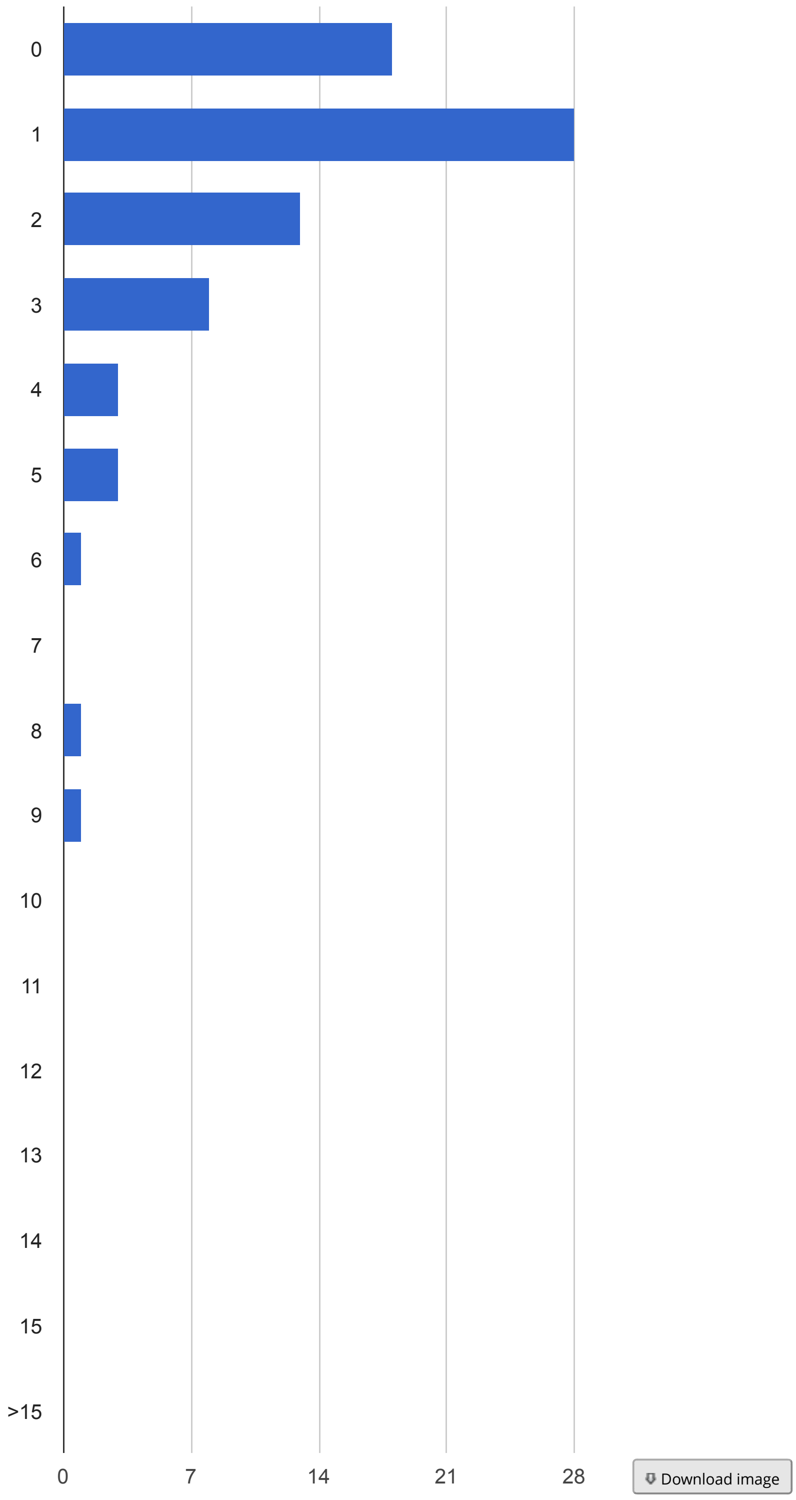

What type are these outbuildings? (check all that apply) *(outbuildings_types)*

Total Count (N)	Missing	Unique
70	8 (10.3%)	5

Counts/frequency: Garage (14, 20.0%), Storage shed (37, 52.9%), Barn (13, 18.6%), Trailer (8, 11.4%), Other (33, 47.1%)

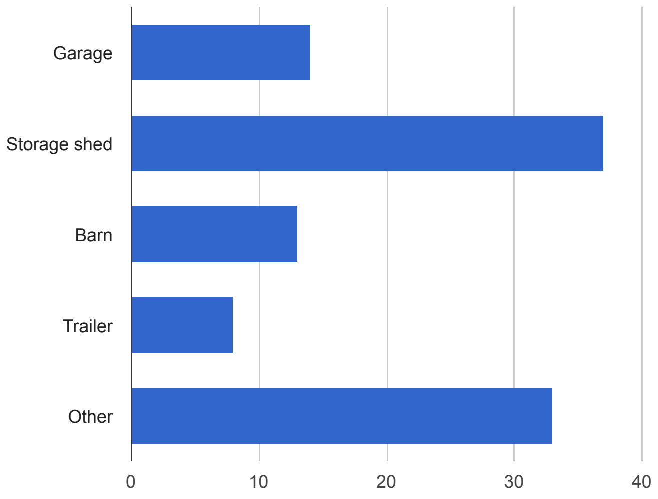

Download image

What "other" type of outbuilding? *(outbuilding_type_other)*

Total Count (N)	Missing
30	48 (61.5%)

Outbuildings Additional Notes: *(outbuildings_add_notes)*

Total Count (N)	Missing
9	69 (88.5%)

Does this property have any of the following: (check all that apply) *(property_misc_items)*

Total Count (N)	Missing	Unique
74	4 (5.1%)	8

Counts/frequency: Woodpile (46, 62.2%), Kennel (5, 6.8%), Chicken Coop (12, 16.2%), Construction Debris (26, 35.1%), Abandoned Car (13, 17.6%), Busted concrete pile (11, 14.9%), Wood or stone bordered beds (36, 48.6%), None (13, 17.6%)

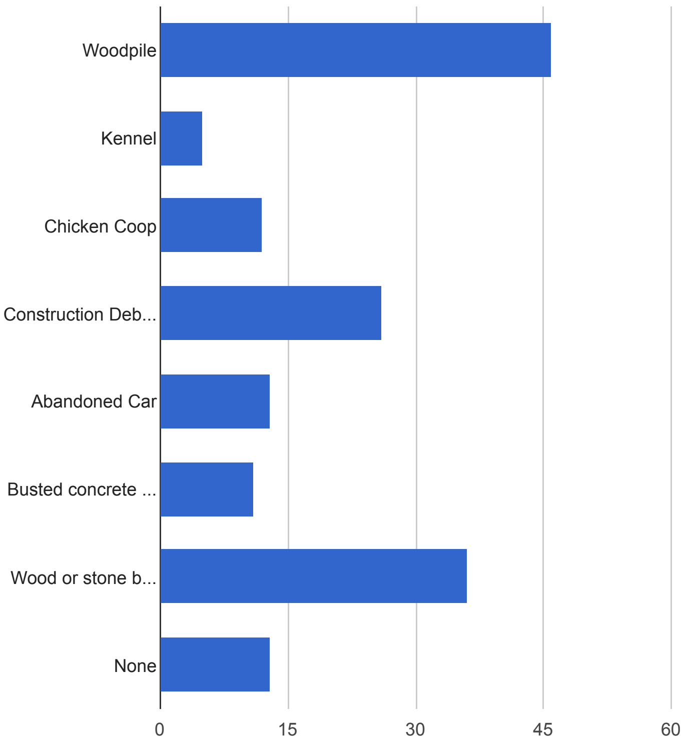

Download image

OTHER Additional Notes: *(property_other_add_notes)*

Total Count (N)	Missing
10	68 (87.2%)

Does the property have landscaping (purposely planted vegetation) on any of the exterior walls of the residence? *(environ_plants_ext_y_n)*

Total Count (N)	Missing	Unique
77	1 (1.3%)	2

Counts/frequency: Yes (35, 45.5%), No (42, 54.5%)

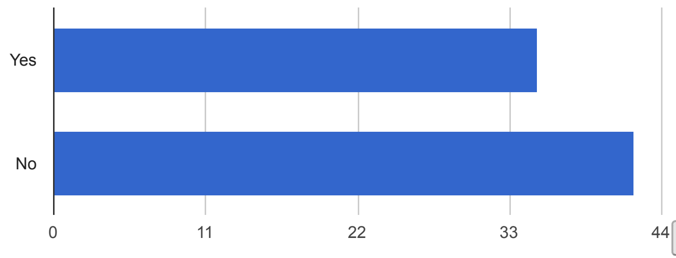

Download image

If "yes", does the landscaping encircle the house? Yes (landscaping encircles house) No (some non-maintained vegetation or landscaping is adjacent to house) *(lanscape_encircle_y_n)*

Total Count (N)	Missing	Unique

35	43 (55.1%)	2

Counts/frequency: Yes (14, 40.0%), No (21, 60.0%)

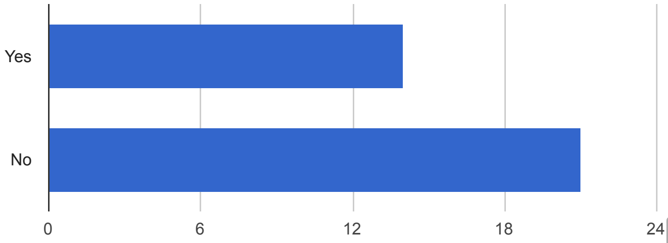

Download image

How well maintained is the landscaping? 2 (well maintained, few weeds) 1 (poorly maintained, over-run with weeds) NA No landscaping *(landscaping_maintained)*

Total Count (N)	Missing	Unique
76	2 (2.6%)	3

Counts/frequency: 2 (52, 68.4%), 1 (5, 6.6%), NA (19, 25.0%)

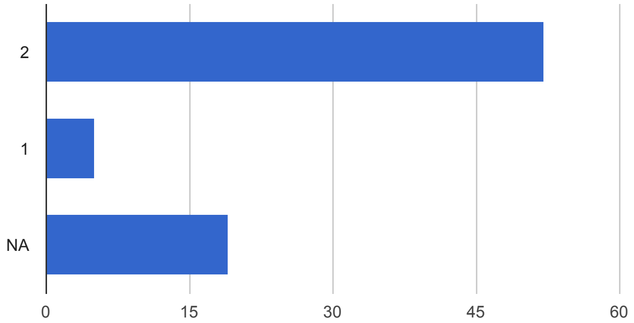

Download image

Survey COMMENTS: *(survey_comments)*

Total Count (N)	Missing
8	70 (89.7%)

Complete? *(tucson_homepersonal_data_collection_instrument_complete)*

Total Count (N)	Missing	Unique
78	0 (0.0%)	2

Counts/frequency: Incomplete (1, 1.3%), Unverified (0, 0.0%), Complete (77, 98.7%)

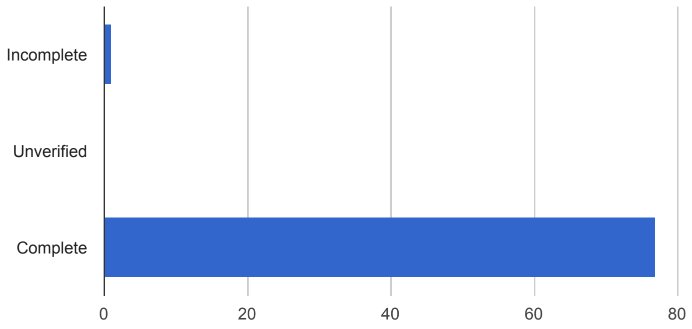

Download image
