# Supplementary material for: Kissing Bug Intrusions into Homes in the Southwest United States
Source: Insects. 2021 Jul 17;12(7):654. doi: 10.3390/insects12070654 (PMC8306929; doi:10.3390/insects12070654)
Supplement: Supplementary file 1 [file insects-12-00654-s001.zip › insects-1278075-Supplement2.pdf]

University of Arizona
Center for Biomedical Informatics & Biostatistics

Tucson Kissing bug Project Home and Personal Evaluation

Data Exports, Reports, and Stats

All data (all records and fields)

Participant ID number (survey ID) *(participant_id)*

Total Count (N)	Missing
94	0 (0.0%)

Age: (years) *(age)*

Total Count (N)	Missing	Unique
93	1 (1.1%)	40

Counts/frequency: 18 (2, 2.2%), 19 (0, 0.0%), 20 (0, 0.0%), 21 (0, 0.0%), 22 (0, 0.0%), 23 (0, 0.0%), 24 (0, 0.0%), 25 (0, 0.0%), 26 (1, 1.1%), 27 (0, 0.0%), 28 (1, 1.1%), 29 (1, 1.1%), 30 (1, 1.1%), 31 (0, 0.0%), 32 (0, 0.0%), 33 (0, 0.0%), 34 (0, 0.0%), 35 (1, 1.1%), 36 (0, 0.0%), 37 (0, 0.0%), 38 (3, 3.2%), 39 (1, 1.1%), 40 (0, 0.0%), 41 (0, 0.0%), 42 (1, 1.1%), 43 (1, 1.1%), 44 (0, 0.0%), 45 (3, 3.2%), 46 (2, 2.2%), 47 (3, 3.2%), 48 (2, 2.2%), 49 (0, 0.0%), 50 (0, 0.0%), 51 (1, 1.1%), 52 (0, 0.0%), 53 (2, 2.2%), 54 (3, 3.2%), 55 (1, 1.1%), 56 (6, 6.5%), 57 (2, 2.2%), 58 (3, 3.2%), 59 (6, 6.5%), 60 (1, 1.1%), 61 (3, 3.2%), 62 (3, 3.2%), 63 (1, 1.1%), 64 (4, 4.3%), 65 (3, 3.2%), 66 (1, 1.1%), 67 (6, 6.5%), 68 (6, 6.5%), 69 (3, 3.2%), 70 (3, 3.2%), 71 (3, 3.2%), 72 (1, 1.1%), 73 (0, 0.0%), 74 (3, 3.2%), 75 (0, 0.0%), 76 (1, 1.1%), 77 (0, 0.0%), 78 (2, 2.2%), 79 (0, 0.0%), 80 (0, 0.0%), 81 (1, 1.1%), 82 (0, 0.0%), 83 (0, 0.0%), 84 (0, 0.0%), 85 (0, 0.0%), 86 (0, 0.0%), 87 (0, 0.0%), 88 (0, 0.0%), 89 (0, 0.0%), 90 (1, 1.1%), 91 (0, 0.0%), 92 (0, 0.0%), 93 (0, 0.0%), 94 (0, 0.0%), 95 (0, 0.0%), 96 (0, 0.0%), 97 (0, 0.0%), 98 (0, 0.0%), 99 (0, 0.0%), 100 (0, 0.0%)

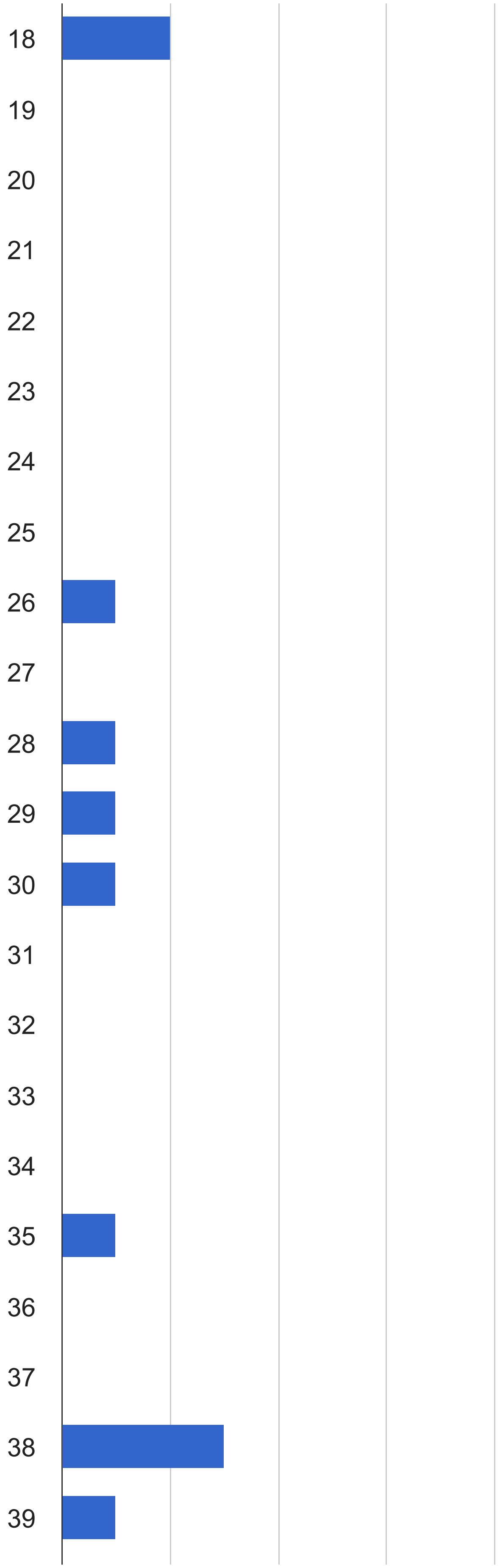
18
19
20
21
22
23
24
25
26
27
28
29
30
31
32
33
34
35
36
37
38
39

Download image

Gender: *(gender)*

Total Count (N)	Missing	Unique
93	1 (1.1%)	2

Counts/frequency: Male (34, 36.6%), Female (59, 63.4%), Do not wish to answer (0, 0.0%)

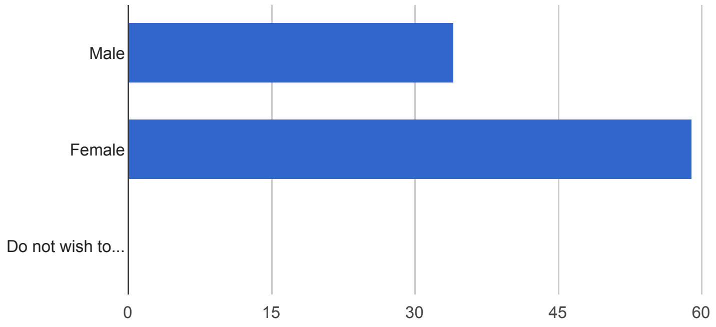

Download image

Zip code: *(zip_code)*

Total Count (N)	Missing
94	0 (0.0%)

1. Have you seen a kissing bug inside your house? *(kb_inside_y_n)*

Total Count (N)	Missing	Unique
94	0 (0.0%)	2

Counts/frequency: Yes (91, 96.8%), No (3, 3.2%)

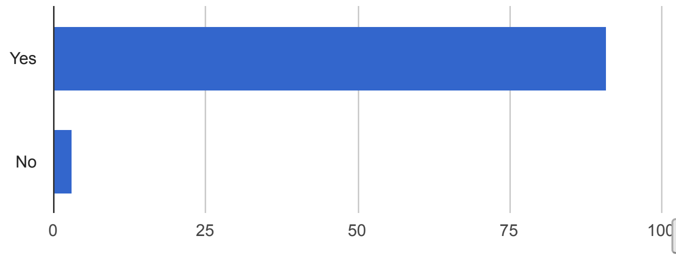

Download image

2. Have you seen a kissing bug outside your house? *(kb_outside_y_n)*

Total Count (N)	Missing	Unique
94	0 (0.0%)	2

Counts/frequency: Yes (64, 68.1%), No (30, 31.9%)

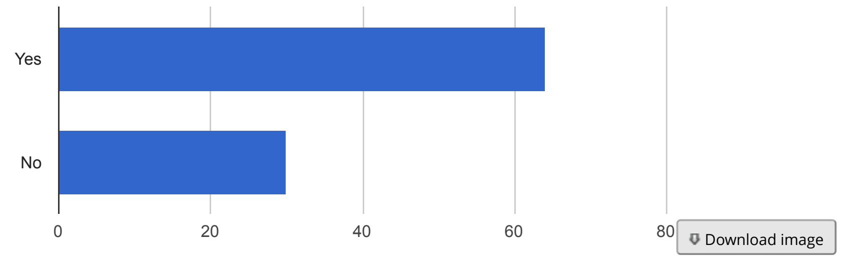

3. Have you been bitten by a kissing bug? *(kb_bitten_y_n)*

Total Count (N)	Missing	Unique
94	0 (0.0%)	2

Counts/frequency: Yes (85, 90.4%), No (9, 9.6%)

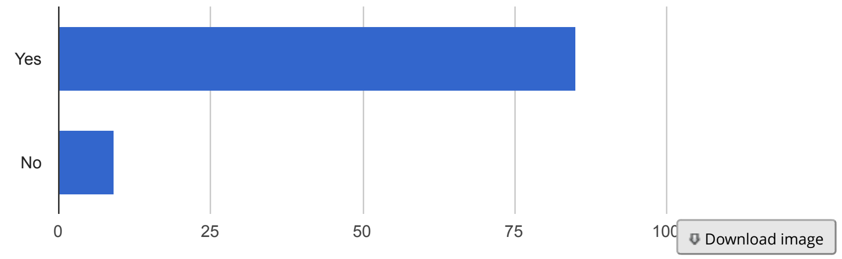

Did it occur while you were sleeping? *(kb_bitten_sleeping_y_n)*

Total Count (N)	Missing	Unique
85	9 (9.6%)	2

Counts/frequency: Yes (77, 90.6%), No (8, 9.4%)

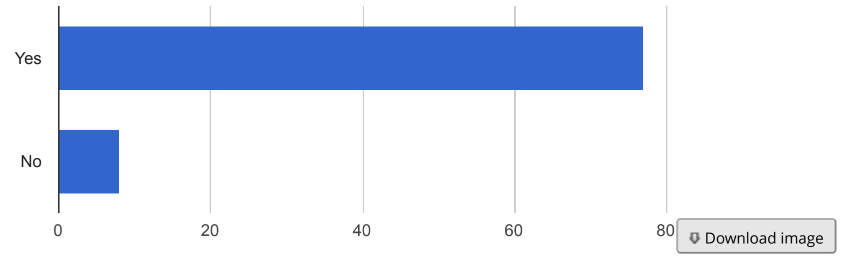

If bitten while you were not sleeping, explain circumstances: *(kb_bitten_not_sleeping)*

Total Count (N)	Missing
8	86 (91.5%)

Insect 1 *(insect_1)*

Total Count (N)	Missing	Unique

94	0 (0.0%)	2

Counts/frequency: Yes (15, 16.0%), No (79, 84.0%)

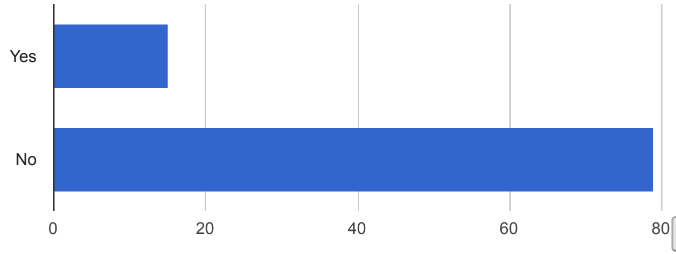

Download image

Insect 2 *(insect_2)*

Total Count (N)	Missing	Unique
94	0 (0.0%)	2

Counts/frequency: Yes (89, 94.7%), No (5, 5.3%)

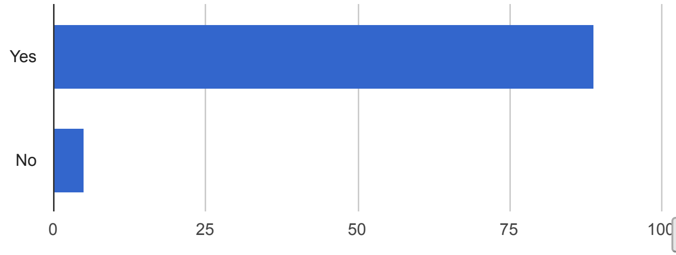

Download image

Insect 3 *(insect_3)*

Total Count (N)	Missing	Unique
94	0 (0.0%)	2

Counts/frequency: Yes (9, 9.6%), No (85, 90.4%)

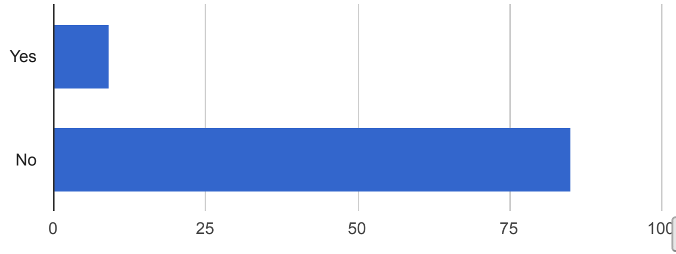

Download image

Insect 4 *(insect_4)*

Total Count (N)	Missing	Unique
94	0 (0.0%)	2

Counts/frequency: Yes (8, 8.5%), No (86, 91.5%)

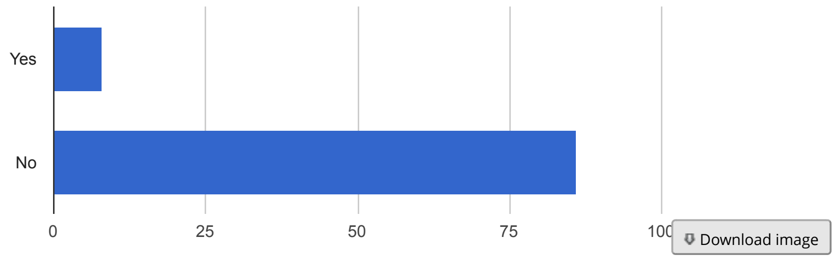

Insect 5 *(insect_5)*

Total Count (N)	Missing	Unique
94	0 (0.0%)	2

Counts/frequency: Yes (23, 24.5%), No (71, 75.5%)

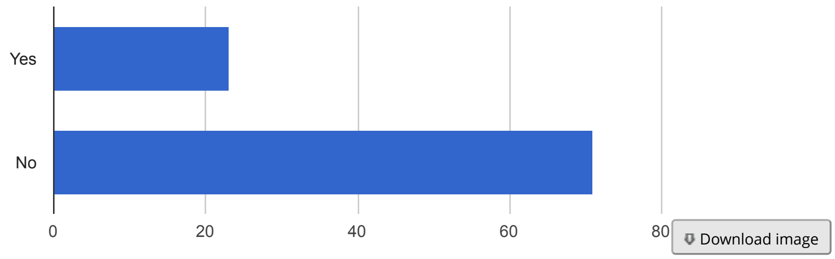

Insect 6 *(insect_6)*

Total Count (N)	Missing	Unique
94	0 (0.0%)	2

Counts/frequency: Yes (27, 28.7%), No (67, 71.3%)

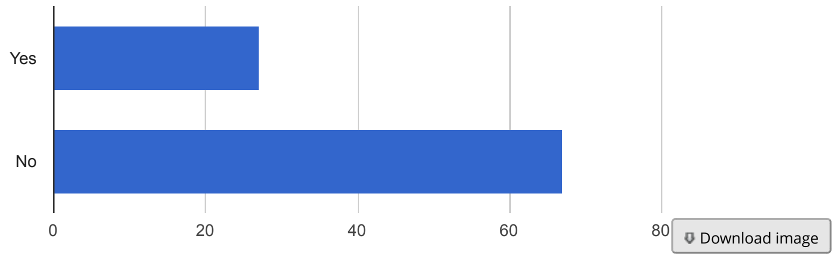

Insect 7 *(insect_7)*

Total Count (N)	Missing	Unique
94	0 (0.0%)	2

Counts/frequency: Yes (88, 93.6%), No (6, 6.4%)

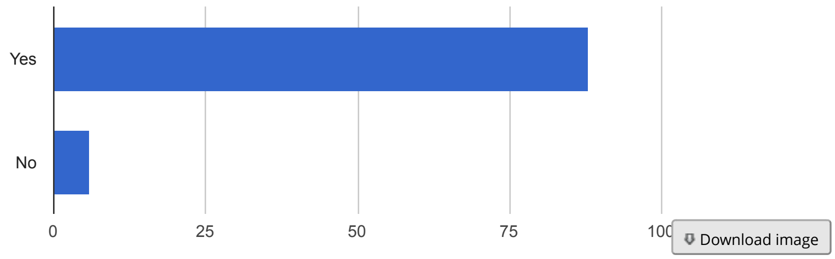

Insect 8 *(insect_8)*

Total Count (N)	Missing	Unique
94	0 (0.0%)	2

Counts/frequency: Yes (76, 80.9%), No (18, 19.1%)

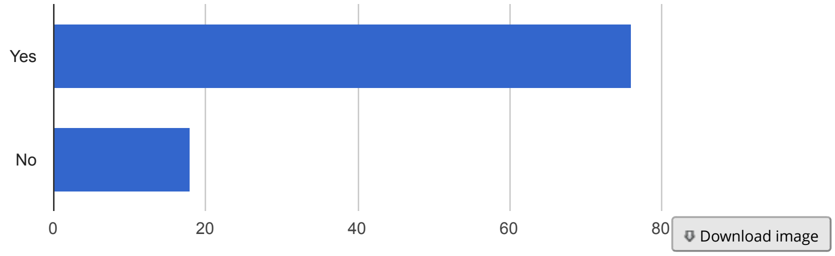

4. Have you changed your sleeping patterns? *(sleeping_patterns_y_n)*

Total Count (N)	Missing	Unique
94	0 (0.0%)	2

Counts/frequency: Yes (33, 35.1%), No (61, 64.9%)

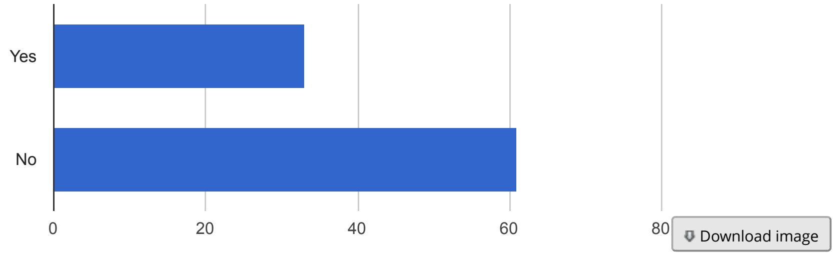

5. Do you sleep under a mosquito net? *(sleep_mosquito_net)*

Total Count (N)	Missing	Unique
94	0 (0.0%)	2

Counts/frequency: Yes (10, 10.6%), No (84, 89.4%)

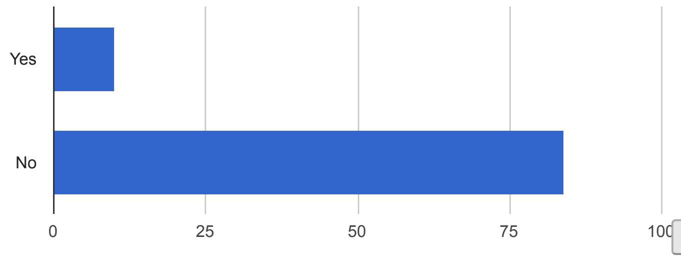

Download image

6. If you were bitten by a kissing bug, did you experience any of the following: (circle all that apply) *(bite_characteristics)*

Total Count (N)	Missing	Unique
91	3 (3.2%)	15

Counts/frequency: Red welt at bite site (73, 80.2%), Pain to touch at bite site (19, 20.9%), Itchiness at bite site (75, 82.4%), Itchiness throughout body (22, 24.2%), Multiple bite sites (63, 69.2%), Dizziness (12, 13.2%), Diffuse skin rash (21, 23.1%), Chest pain (4, 4.4%), Swelling at bite site (56, 61.5%), Swollen lips (4, 4.4%), Swollen face (6, 6.6%), Swollen tongue (5, 5.5%), Shortness of breath (difficulty breathing) (13, 14.3%), Feeling of "uneasiness" (23, 25.3%), None (12, 13.2%)

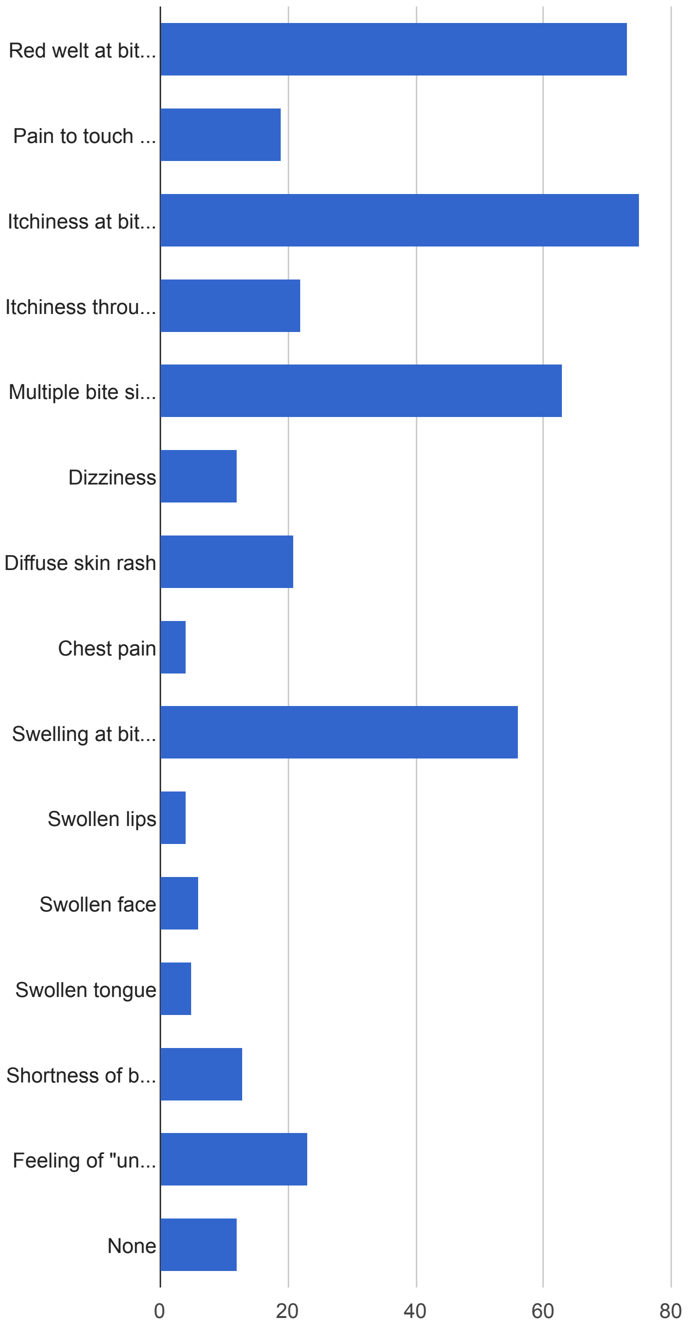

Download image

7. What sites have been bitten before on your body? (check all that apply) *(bite_regions)*

Total Count (N)	Missing	Unique
91	3 (3.2%)	8

Counts/frequency: Face (17, 18.7%), Neck (28, 30.8%), Arms (53, 58.2%), Legs (54, 59.3%), Back (37, 40.7%), Hands (17, 18.7%), Feet (13, 14.3%), Other regions... (33, 36.3%)

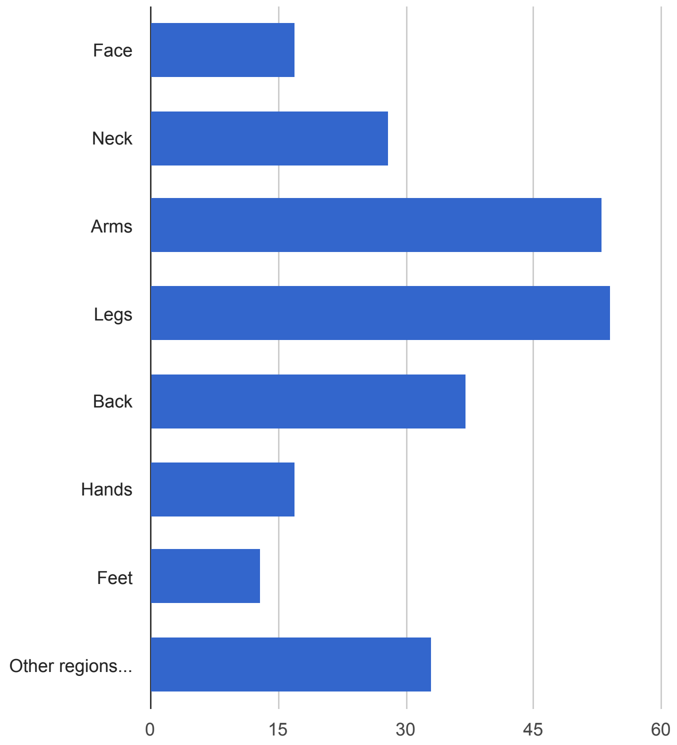

Download image

What "other regions" were bitten? *(bite_other_regions)*

Total Count (N)	Missing
32	62 (66.0%)

8. What intervention did you take after being bitten? (check all that apply) *(bite_intervention)*

Total Count (N)	Missing	Unique
90	4 (4.3%)	8

Counts/frequency: Called 911 (went to emergency room) (3, 3.3%), Called Poison Control Center (2, 2.2%), Washed bite site (30, 33.3%), Put lotion on bite site (6, 6.7%), Applied herbal topical lotion (8, 8.9%), Applied topical medicine: (such as anti-itch cream) (35, 38.9%), I did nothing after I was bitten (35, 38.9%), Other intervention... (20, 22.2%)

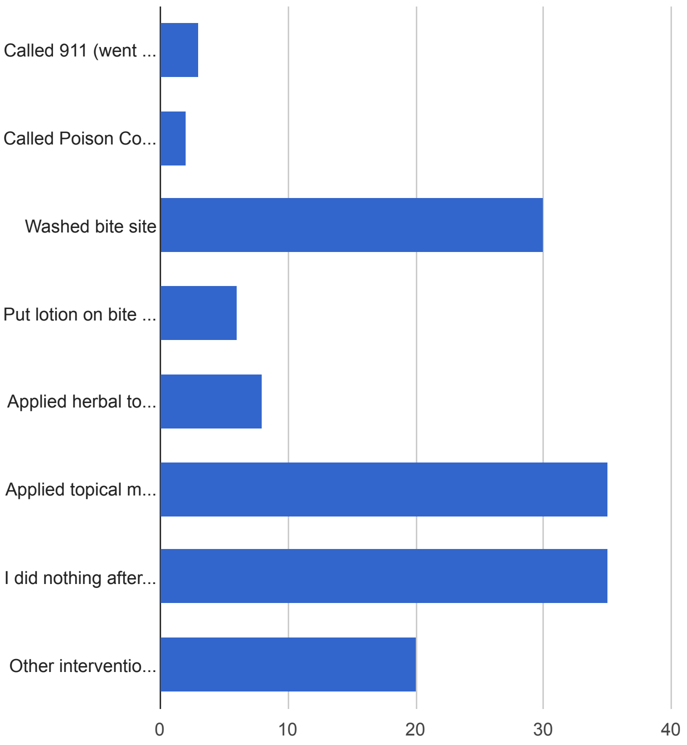

Download image

What "other intervention" did the participant do? *(bite_intervention_other)*

Total Count (N)	Missing
20	74 (78.7%)

Did the participant take over the counter medication? *(bite_otc_y_n)*

Total Count (N)	Missing	Unique
92	2 (2.1%)	2

Counts/frequency: Yes (34, 37.0%), No (58, 63.0%)

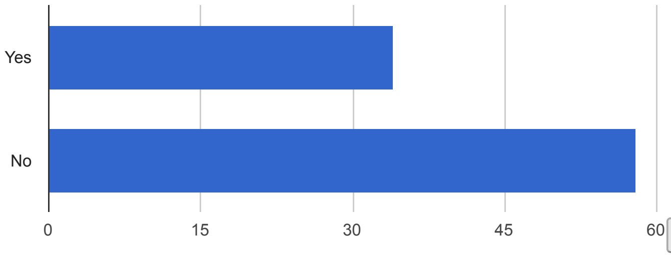

Download image

Which over the counter medication(s)? (check all that apply)
(otc_which_meds)

Total Count (N)	Missing	Unique
34	60 (63.8%)	5

Counts/frequency: Aspirin (1, 2.9%), Tylenol (2, 5.9%), Ibuprofen (Motrin) (3, 8.8%), Benadryl (28, 82.4%), Other medications... (3, 8.8%)

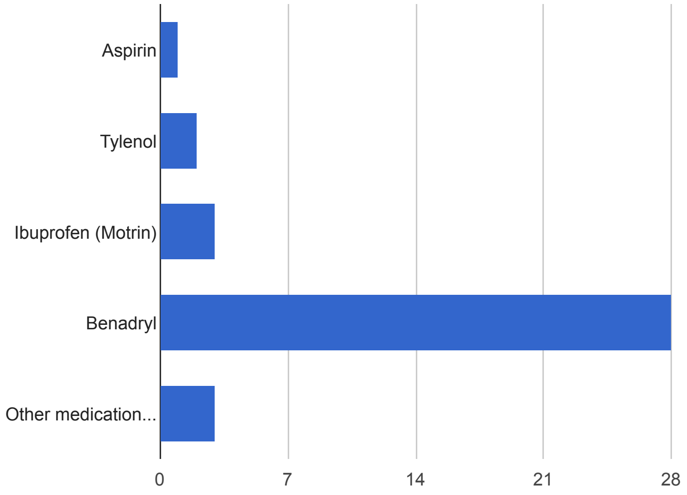

Download image

What "other medication(s)" did the participant take? *(otc_med_other)*

Total Count (N)	Missing
3	91 (96.8%)

9. How many times have you been bitten by a kissing bug? (only one answer) *(bitten_how_many_times)*

Total Count (N)	Missing	Unique
90	4 (4.3%)	6

Counts/frequency: Once (11, 12.2%), 2-4 (22, 24.4%), 5-10 (23, 25.6%), 11-50 (31, 34.4%), 51-100 (1, 1.1%), >100 (2, 2.2%)

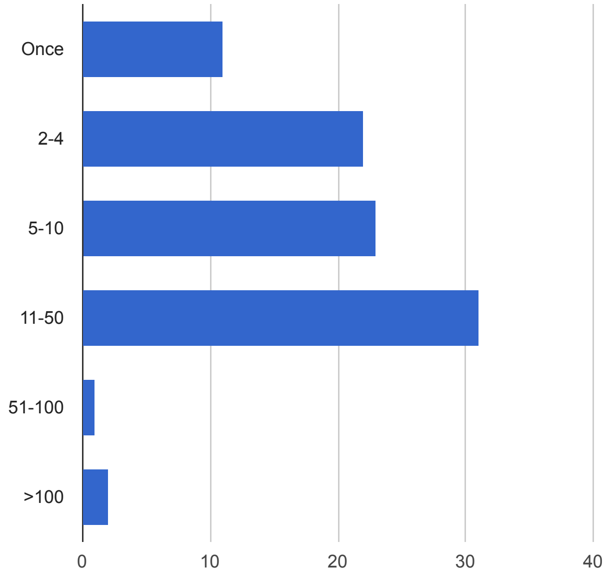

Download image

10. Do you have a known anaphylaxis to a kissing bug bite?

(bite_anaphylaxis_y_n)

Total Count (N)	Missing	Unique
94	0 (0.0%)	2

Counts/frequency: Yes (9, 9.6%), No (85, 90.4%)

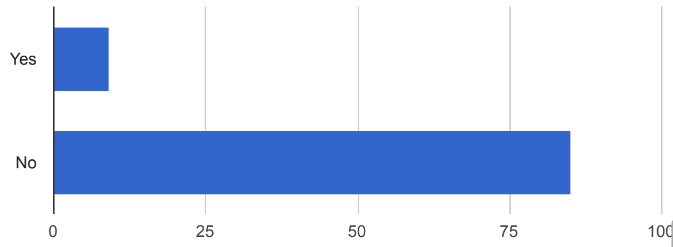

Download image

If so, do they have an epinephrine autoinjector (pen) to use in case you are bitten again? *(anaphylaxis_epi_pen_y_n)*

Total Count (N)	Missing	Unique
9	85 (90.4%)	2

Counts/frequency: Yes (6, 66.7%), No (3, 33.3%)

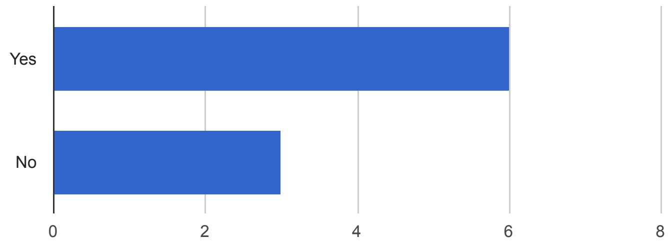

Download image

11. How many episodes of anaphylaxis have you had due to a kissing bug bite? (circle one) *(anaphylaxis_episodes)*

Total Count (N)	Missing	Unique
94	0 (0.0%)	5

Counts/frequency: None (84, 89.4%), Once (6, 6.4%), 2 (1, 1.1%), 3 (1, 1.1%), 4 (0, 0.0%), 5 (0, 0.0%), 6 (0, 0.0%), 7 (0, 0.0%), 8 (0, 0.0%), 9 (0, 0.0%), 10 (0, 0.0%), >10 (2, 2.1%)

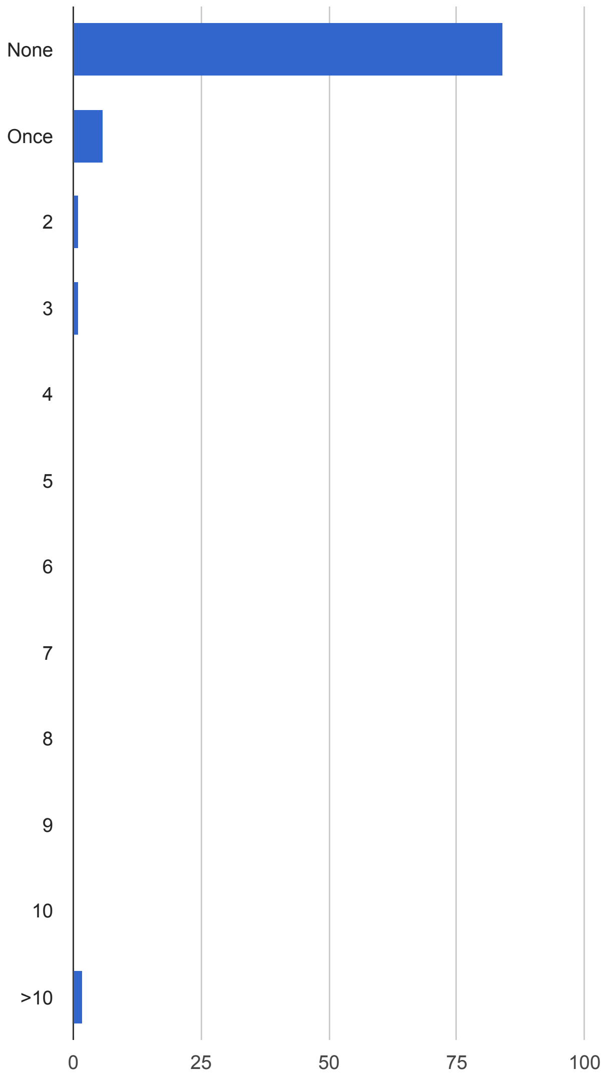

Download image

12. If you ever sought medical attention after a kissing bug bite, did you feel the provider had knowledge of kissing bug bites?

(bite_provider_knowledge)

Total Count (N)	Missing	Unique
94	0 (0.0%)	3

Counts/frequency: Yes (14, 14.9%), No (27, 28.7%), Does not apply (have not seen a provider) (53, 56.4%)

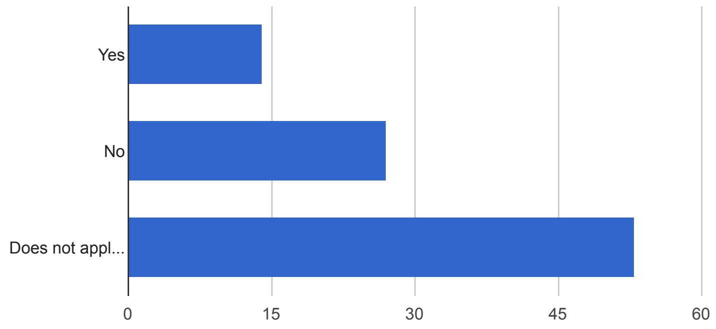

Download image

13. Knowing kissing bugs bite at night, how safe do you feel at

bedtime? (1 being least safe and 10 being most unsafe) *(bite_how_safe)*

Total Count (N)	Missing	Unique
94	0 (0.0%)	10

Counts/frequency: 1 (9, 9.6%), 2 (6, 6.4%), 3 (11, 11.7%), 4 (3, 3.2%), 5 (16, 17.0%), 6 (5, 5.3%), 7 (12, 12.8%), 8 (11, 11.7%), 9 (7, 7.4%), 10 (14, 14.9%)

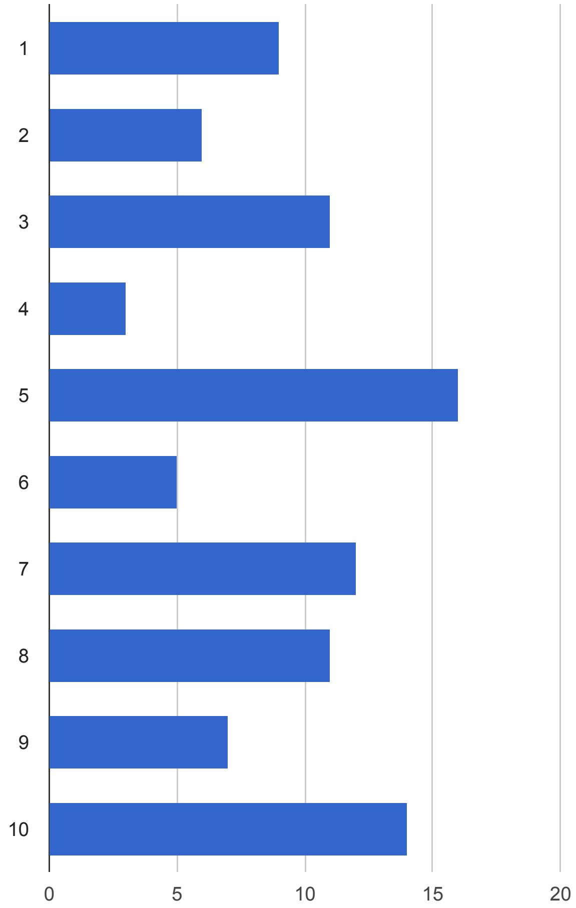

Download image

14. How afraid of kissing bugs (scale of 1 - 10)? (1 being not afraid and 10 being most afraid) *(kissing_bug_afraid)*

Total Count (N)	Missing	Unique
94	0 (0.0%)	10

Counts/frequency: 1 (22, 23.4%), 2 (11, 11.7%), 3 (8, 8.5%), 4 (8, 8.5%), 5 (9, 9.6%), 6 (4, 4.3%), 7 (7, 7.4%), 8 (12, 12.8%), 9 (2, 2.1%), 10 (11, 11.7%)

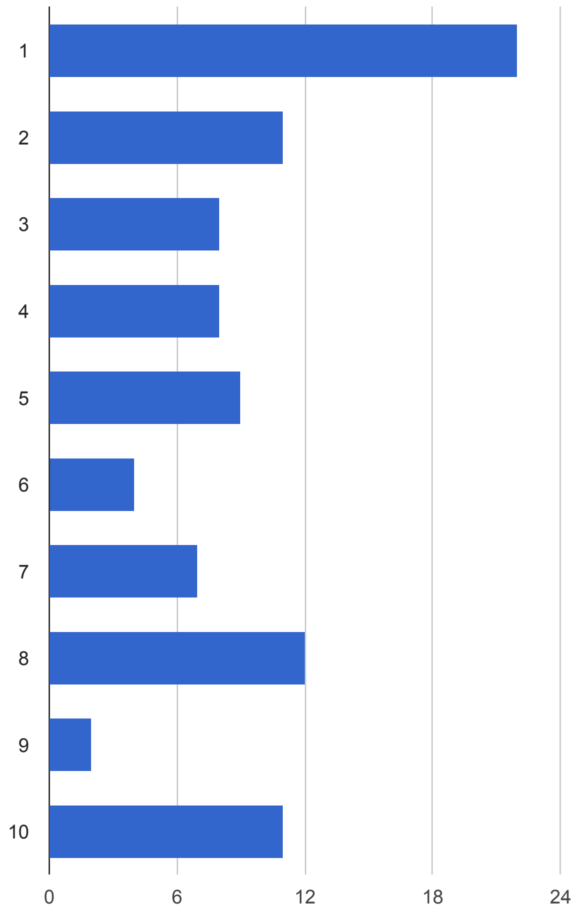

Download image

15. How frustrated are you with kissing bugs? (scale of 1 - 10) (1 being not frustrated and 10 being extremely frustrated) *(kissing_bug_frustrated)*

Total Count (N)	Missing	Unique
94	0 (0.0%)	10

Counts/frequency: 1 (8, 8.5%), 2 (9, 9.6%), 3 (5, 5.3%), 4 (3, 3.2%), 5 (6, 6.4%), 6 (5, 5.3%), 7 (12, 12.8%), 8 (12, 12.8%), 9 (7, 7.4%), 10 (27, 28.7%)

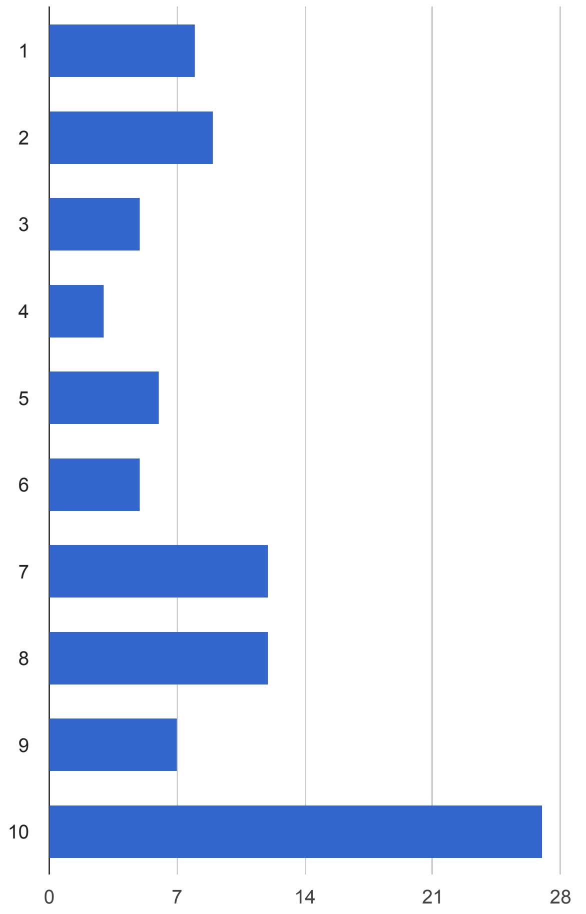

Download image

Did participant leave comments at the end? *(survey_end_comments_y_n)*

Total Count (N)	Missing	Unique
94	0 (0.0%)	2

Counts/frequency: Yes (22, 23.4%), No (72, 76.6%)

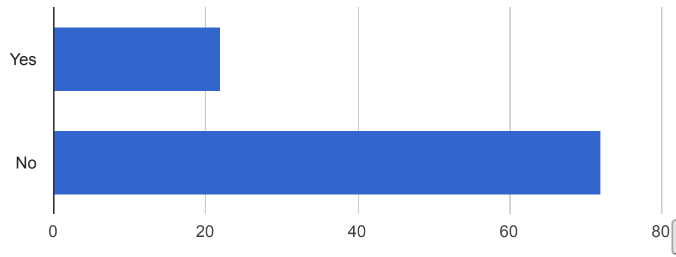

Download image

Comments: *(survey_end_comments)*

Total Count (N)	Missing
22	72 (76.6%)

Complete? *(kissing_bug_bite_survey_complete)*

Total Count (N)	Missing	Unique
94	0 (0.0%)	1

Counts/frequency: Incomplete (0, 0.0%), Unverified (0, 0.0%), Complete (94, 100.0%)

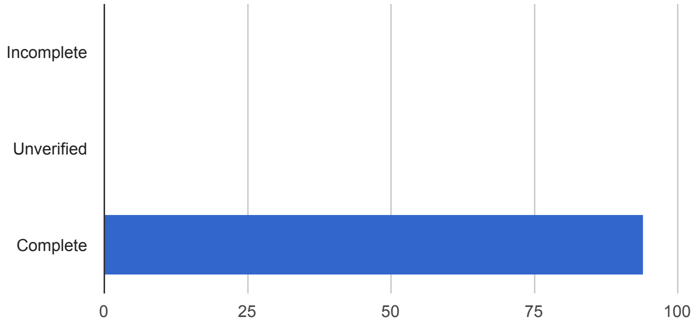

Download image
